# Development and technical characterization of a biomimetic thorax simulator for regional intrapulmonary pressure measurement

**DOI:** 10.3389/fbioe.2026.1907500

**Published:** 2026-10-22

**Authors:** Keywan Sohrabi, Jasmin Spaar, Tibor Jung, Niklas Ostwald, Patrick Fischer, Oskar Seifert, Dominik Kraft, István Vadász

**Affiliations:** 1 Faculty of Health Sciences, University of Applied Sciences, Giessen, Germany; 2 Medical Faculty, Justus Liebig University, Giessen, Germany; 3 Institute of Medical Informatics (IMI), Justus-Liebig-University, Giessen, Germany; 4 Faculty of Veterinary Medicine, Small Animal Clinic, Justus Liebig University, Giessen, Germany; 5 Department of Internal Medicine, Universities of Giessen and Marburg Lung Center, German Center for Lung Research (DZL), Giessen, Germany; 6 Institute for Lung Health (ILH), Giessen, Germany; 7 Excellence Cluster Cardio-Pulmonary Institute (CPI), Giessen, Germany

**Keywords:** *ex vivo*, intrapulmonary pressure measurement, lung, mechanical ventilation (MV), PEEP, thorax simulator

## Abstract

**Background:**

Mechanical ventilation is a cornerstone therapy for respiratory failure, but heterogeneous regional pressure distribution can lead to localized lung stress and ventilator-induced lung injury. To accurately investigate these dynamics, physiologically realistic experimental platforms are required. However, current experimental models either rely on simplified mechanical lung analogues or lack the capability to measure spatially resolved intrapulmonary pressure dynamics.

**Methods:**

A biomimetic thorax–lung simulator incorporating an *ex vivo* porcine lung was used to measure regional intrapulmonary pressures during pressure-controlled mechanical ventilation. The lung was positioned within an airtight thoracic chamber operated at a nominal subatmospheric baseline pressure and connected to an anatomically realistic upper airway model. Pressure signals were recorded simultaneously at seven anatomical locations, including the trachea, main bronchi, and upper and lower lung regions. Ventilation was performed across positive end-expiratory pressure (PEEP) levels ranging from 0 to 20 mbar while maintaining a constant driving pressure of 15 mbar. Regional pressure transmission, pressure-trajectory hysteresis, and Poincaré-derived dispersion were analyzed together with tidal volume and dynamic system compliance.

**Results:**

The platform enabled simultaneous acquisition of regional intrapulmonary pressure signals, airway pressure, flow, and chamber pressure across all investigated ventilator settings. Repeated measurements showed high within-specimen technical repeatability, with repeated median regional pressure values differing by approximately 0.02 mbar. Regional pressure attenuation, pressure-flow indices, and trachea-to-region regression parameters were derived as engineering metrics of pressure transmission. Tidal volume and dynamic system compliance were reconstructed from the synchronized flow and tracheal-pressure signals. In the investigated specimen, peripheral regions showed greater pressure-trajectory hysteresis and pressure-state dispersion at low PEEP than under higher PEEP conditions.

**Conclusion:**

The developed platform enables synchronized regional intrapulmonary pressure measurements under controlled mechanical ventilation and showed high short-term within-specimen repeatability. In the investigated specimen, static pressure transmission was largely homogeneous, whereas regional pressure-trajectory dynamics differed between central and peripheral measurement sites, particularly at low PEEP. Increasing PEEP was associated with reduced regional pressure-trajectory hysteresis and variability within this specimen. These findings demonstrate the technical feasibility of the platform and provide a basis for subsequent validation in additional specimens and against independent physiological reference data.

## 1 Introduction

Mechanical ventilation is a cornerstone therapy for patients with acute or chronic respiratory failure; however, mechanical stress and strain may contribute to ventilator-induced lung injury. The mechanical forces acting on the lung during ventilation depend not only on ventilator settings but also on the interaction between airway pressure, pleural pressure, and the mechanical properties of the thorax and lung. The distending force applied to lung tissue is the transpulmonary pressure, defined as the difference between alveolar and pleural pressure, and it represents a key determinant of lung inflation and lung mechanics (Acute Respiratory Distress Syndrome Network et al., 2000; Protti et al., 2013; Talmor et al., 2008; Yoshida et al., 2018).

Lung inflation is not spatially uniform. Regional transpulmonary pressure gradients arise from gravitational forces, anatomical differences, and mechanical heterogeneity of the thorax and lung system (Protti et al., 2013; Talmor et al., 2008). Experimental measurements in both animals and humans demonstrate considerable spatial variability of pleural pressure and lung stress. These variations contribute to uneven ventilation distribution and can lead to localized regions of overdistension or collapse within the lung parenchyma (Beitler et al., 2019; Mead et al., 1970; Terzi et al., 2020; Wellman et al., 2012).

These regional variations in transpulmonary pressure and lung stress provide the physiological basis for protective ventilation strategies, which aim to limit mechanical stress and strain in order to mitigate ventilator-induced lung injury. The Acute Respiratory Distress Syndrome Network trial established low tidal volume ventilation as the clinical standard of care for patients with acute respiratory distress syndrome (ARDS) (Acute Respiratory Distress Syndrome Network et al., 2000; Amato et al., 2015). Subsequent studies identified driving pressure and mechanical power as important determinants of ventilator induced lung injury (Carvalho et al., 2011; D’Albo et al., 2024; Gattinoni et al., 2016a; Zaidi et al., 2024). Driving pressure reflects the pressure required to inflate the respiratory system during tidal ventilation, while mechanical power integrates several ventilatory variables into a global estimate of the energy delivered to the lung (Hoppe et al., 2023).

Although these parameters are clinically valuable, they are derived from global airway measurements and therefore do not capture the regional distribution of pressure and stress within the lung. In heterogeneous lungs, particularly in ARDS, large spatial differences in ventilation and tissue stress can occur. Consequently, global respiratory mechanics may not adequately reflect the mechanical conditions experienced by individual lung regions.

Measurement of esophageal pressure has been introduced as a clinical method to estimate pleural pressure and to guide titration of positive end expiratory pressure and transpulmonary pressure targets (Fish et al., 2014; Florio et al., 2024; Suarez-Sipmann et al., 2019; Yoshida et al., 2018). While this technique provides useful physiological information, esophageal pressure measurements reflect pleural conditions only in a limited region adjacent to the esophagus. Experimental and translational studies indicate that these measurements may not accurately represent pressure conditions in dependent or peripheral regions of the lung, especially in diseased or anatomically complex situations (Franchi et al., 2024; Olivieri et al., 2012; Terzi et al., 2020). Direct measurement of regional intrapulmonary pressure therefore represents an important but rarely implemented experimental approach.

Bench testing using active lung simulators has significantly improved the evaluation of ventilator performance and patient ventilator interaction under controlled conditions. Devices such as the ASL 5000 allow simulation of respiratory effort, lung compliance, and airway resistance and are widely used to investigate ventilator triggering, leak compensation, and patient ventilator synchrony (Dexter et al., 2018; Judge et al., 2014; Lebret et al., 2022; Martínez-Castro et al., 2023). However, these simulators rely on simplified mechanical lung analogues and therefore cannot reproduce the anatomical structure, airway branching patterns, or heterogeneous mechanical behavior of biological lungs.

From an engineering perspective, current ventilator test systems provide high controllability but limited anatomical realism, whereas biological *ex vivo* preparations preserve airway geometry and tissue mechanics but are often less standardized in instrumentation and signal acquisition. A hybrid experimental platform combining biological lung tissue with controlled thoracic boundary conditions and synchronized regional pressure acquisition may help bridge the gap between conventional bench simulators and physiological lung models.

*Ex vivo* porcine lungs represent a translationally relevant model for respiratory research. The porcine lung closely resembles human lung anatomy in airway branching, lobar structure, and overall size (Castro et al., 2020; Kamusella et al., 2009). *Ex vivo* lung preparations obtained from slaughterhouse tissue or isolated perfused lung systems have been widely used to study respiratory mechanics, ventilation strategies, and pulmonary pathology under controlled experimental conditions (Linton, 2005; Semenov et al., 2024; Vadász et al., 2005). These models preserve realistic tissue compliance and airway geometry while avoiding ethical and physiological variability associated with *in vivo* experiments.

Nevertheless, most *ex vivo* lung studies employ positive pressure ventilation without physiologic thoracic boundary conditions and typically measure only airway pressure or global respiratory mechanics. Direct measurements of regional intrapulmonary pressure within such models remain scarce.

To address these limitations, an experimental system should combine biological airway anatomy with defined and reproducible thoracic boundary conditions. Physiological spontaneous breathing is associated with subatmospheric pleural pressure generated by respiratory muscle activity, and negative-pressure ventilation systems demonstrate that external thoracic pressure can substantially influence lung inflation and respiratory mechanics (Forgie et al., 2025; Thomasian and Wunsch, 2025; Vartazarmian et al., 2026). Experimental *ex vivo* platforms have also used controlled negative-pressure environments to investigate lung mechanics under defined boundary conditions (Buchko et al., 2020; Quiros et al., 2024).

The present system was not designed to reproduce the physiological pleural-pressure waveform or spontaneous negative-pressure breathing. Instead, conventional positive-pressure mechanical ventilation was combined with a controlled subatmospheric extrathoracic baseline condition. This configuration provides a reproducible experimental environment in which regional intrapulmonary pressure transmission can be measured directly while preserving biological airway geometry.

The aim of this study was therefore to establish and technically characterize a biomimetic thorax–lung simulator for synchronized measurement of regional intrapulmonary pressures during mechanical ventilation. We investigated regional pressure transmission across different PEEP levels, quantified pressure-trajectory hysteresis and pressure-state dispersion, and assessed within-specimen repeatability as well as global ventilation-derived metrics. The study was designed as a technical feasibility investigation using a single lung specimen and was not intended to establish generalized physiological responses or to validate PEEP-related treatment strategies.

## 2 Materials and methods

### 2.1 General methodology and workflow

To evaluate regional intrapulmonary pressure dynamics and overall system performance, a structured experimental workflow was implemented comprising three main steps: (1) preparation and integration of an *ex vivo* porcine lung into the biomimetic thorax simulator; (2) establishment of a synchronized multi-sensor measurement chain for regional pressure, airway pressure, airflow, and chamber pressure; and (3) application of controlled mechanical ventilation across different PEEP levels to assess technical feasibility, regional pressure transmission, and within-specimen repeatability. Signal processing included temporal synchronization, automated respiratory-phase detection, regional pressure and pressure-flow analysis, reconstruction of tidal volume and dynamic compliance, and exploratory pressure-trajectory analyses.

### 2.2 Experimental setup and study design

This study was conducted as a technical feasibility and system-characterization investigation using a single *ex vivo* porcine lung specimen. The use of one specimen was intentional because the primary objective was to evaluate the integrated measurement platform under consistent anatomical and mechanical conditions rather than to quantify biological inter-specimen variability. Each ventilation condition was repeated three times to assess short-term technical within-specimen repeatability. Validation across multiple specimens and against independent physiological reference data remains necessary.

Experiments were performed using a custom biomimetic thorax–lung simulator (Figure 1). The system consisted of an airtight thoracic chamber containing the *ex vivo* porcine lung, an anatomically realistic upper airway model, an endotracheal airway interface, a mechanical ventilator, a multichannel regional pressure measurement system, an inline flow and pressure analyzer, and a vacuum pump.

**FIGURE 1:**
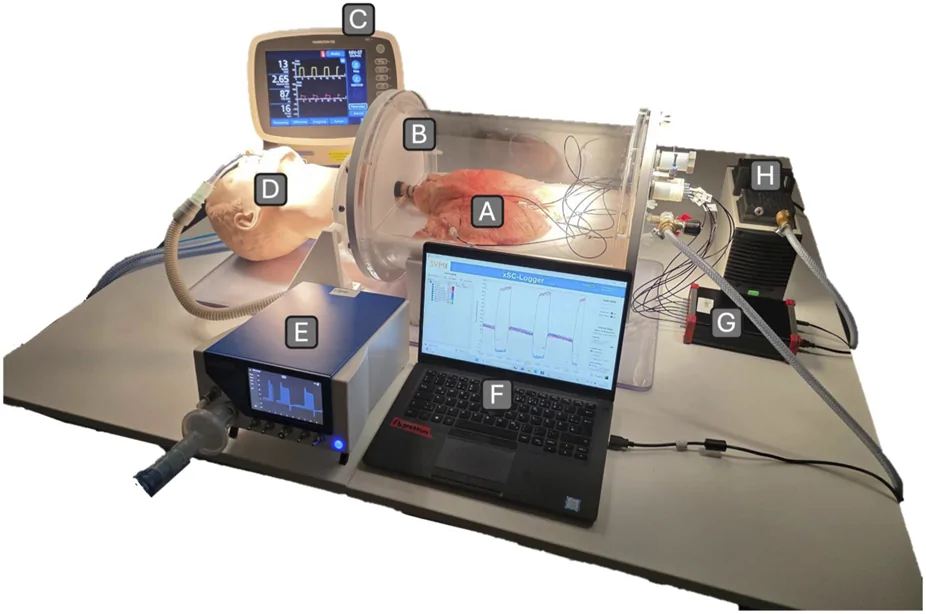
Setup of the biomimetic thorax-lung simulator for the experiments with **(A)** porcine lung, **(B)** biomimetic thorax, **(C)** Ventilator Hamilton C2, **(D)** biomimetic head, **(E)** gas flow meter FlowAnalyser PF300Pro, **(F)** Notebook with measurement device control software, **(G)** pressure scanner PSC8, **(H)** vacuum pump.

Mechanical ventilation was generated by the Hamilton C2 ventilator, whereas the thoracic chamber was operated at a nominal subatmospheric pressure of approximately −7 mbar using a controllable vacuum pump (N 938.50 KT.18 LABOPORT, KNF DAC GmbH, Germany). The chamber pressure therefore represented a controlled extrathoracic engineering boundary condition and was not intended to reproduce a physiological pleural-pressure waveform. No actuated diaphragm analogue was used in the experiments reported in this study.

The trachea was connected through a sealed adapter to an upper airway model representing nasal, oral, pharyngeal, and glottic structures. This configuration enabled simultaneous measurement of regional intrapulmonary pressures in central and peripheral lung regions during controlled positive-pressure mechanical ventilation.

### 2.3 *Ex vivo* lung preparation and integration into the thorax simulator

A preserved porcine lung specimen (Nasco Inflatable Lung Kit, Nasco Education, USA) was used as the *ex vivo* lung model. According to the manufacturer, the tissue originates from animals processed within the regular food-production chain and is preserved to maintain anatomical structure and elastic mechanical properties.

Before integration into the simulator, the specimen underwent macroscopic inspection for structural integrity. Airtightness was assessed by applying low airway pressure through the tracheal connector and verifying pressure stability and the absence of visible macroscopic leakage.

The lung was positioned within the thoracic chamber while preserving its cranio-caudal orientation and bronchial branching geometry. The trachea was connected to the airway interface through a sealed connector and an endotracheal tube (InterTube, Well Lead Medical Co. Ltd.; internal diameter 8.0 mm), thereby maintaining separation between the intrapulmonary and chamber-pressure compartments.

### 2.4 Multi-sensor measurement chain

#### 2.4.1 Intrapulmonary pressure measurement

Regional intrapulmonary pressure was measured at seven anatomical locations (Figure 2):

**FIGURE 2:**
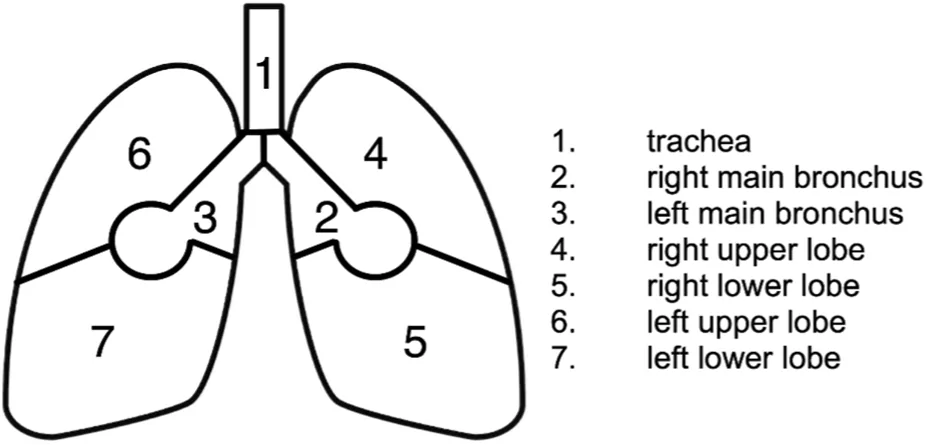
Anatomical locations of regional pressure measurements.

Pressure sensing lines made from polyethylene with an internal diameter of 1 mm and a length of 60 cm were connected to a multichannel pressure scanner (PSC8, SVMtec, Germany). The regional pressure lines were introduced using single-use 26-G cannulas with a length of 25 mm. Because airway dimensions differed between anatomical locations, no uniform insertion depth was applied. Cannulas were positioned individually according to local airway anatomy, with the opening located as centrally as possible within the respective airway lumen.

Before each measurement sequence, all pressure channels were exposed to a common pressure condition to verify baseline consistency and unobstructed pressure transmission. During placement, a constant airflow condition was additionally used to identify obstruction or inconsistent channel behavior. Repeated measurements under unchanged experimental conditions were used to assess signal stability and technical repeatability.

No independent multi-point recalibration of the pressure scanner was performed as part of this study, absolute pressure measurements relied on the calibration specification of the commercial measurement system.

#### 2.4.2 Instrumentation and data acquisition

Regional intrapulmonary pressures were measured using a PSC8 multichannel pressure scanner (SVMtec, Germany) with a measurement range of ±350 mbar and a specified accuracy of 0.1% of full-scale span. Pressure signals were digitized at a sampling frequency of 100 Hz, corresponding to a sampling interval of 0.01 s, and stored in TDMS format.

Airway pressure and airflow were recorded simultaneously using a FlowAnalyser PF 300 PRO (IMT Analytics, Switzerland) positioned inline between the ventilator and the airway model. The FlowAnalyser generated independent LOG files containing pressure and airflow signals.

The resulting raw dataset therefore comprised (i) seven regional intrapulmonary pressure channels, (ii) airway pressure and airflow, and (iii) chamber-pressure recordings. TDMS and FlowAnalyser recordings were subsequently synchronized computationally before phase-specific and ventilation-derived metrics were calculated (Figure 3).

**FIGURE 3:**
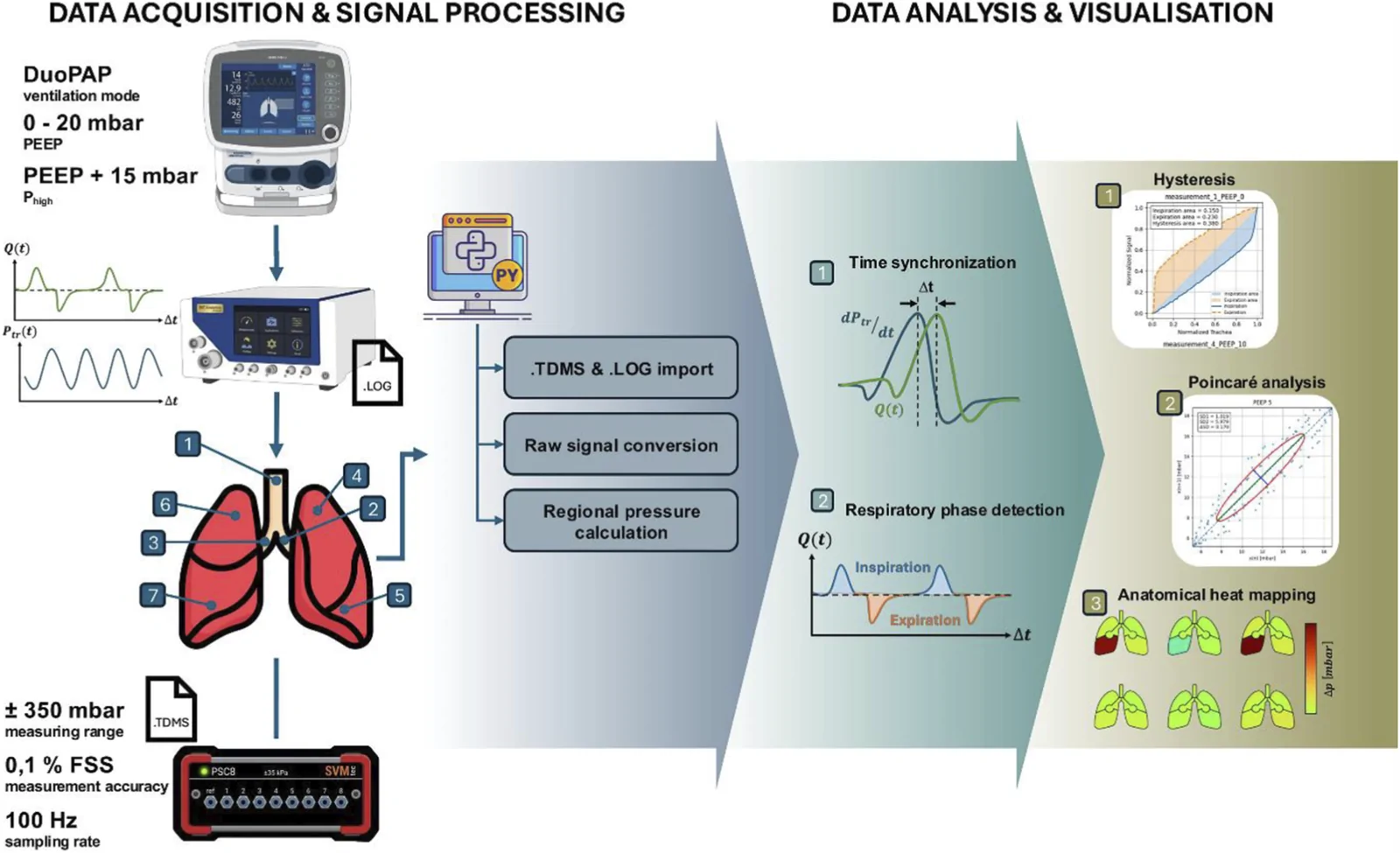
Schematic overview of the experimental setup and data-processing workflow. **Data acquisition** included regional intrapulmonary pressure measurements, tracheal pressure, airflow, and chamber pressure. **Signal preprocessing** included data import, pressure scaling, temporal interpolation, and cross-correlation-based synchronization. **Data analysis** included automated respiratory-phase detection, regional pressure and pressure-flow analysis, reconstruction of tidal volume and dynamic compliance, pressure-trajectory hysteresis analysis, and Poincaré-derived dispersion metrics. Visualization included regional anatomical pressure maps, pressure-trajectory plots, and descriptive comparison of regional metrics.

### 2.5 Ventilation protocol

Ventilation was performed using a Hamilton C2 (C3SN 7213, Hamilton Medical AG, Switzerland) operating in DuoPAP mode, a pressure-controlled ventilation mode alternating between two airway pressure levels, P_high_ and PEEP. Starting from PEEP 0 mbar, PEEP was increased stepwise to 5, 7, 15, and 20 mbar while maintaining a constant driving pressure (P_high_ − PEEP) of 15 mbar. After completion of the PEEP 20 condition, PEEP and P_high_ were returned to 0 and 15 mbar, respectively, for a final PEEP 0 condition to assess the effect of prior high-PEEP exposure (Table 1).

**TABLE 1:** Ventilation protocol.

Measurement	Mode	PEEP (mbar)	P_high_ (mbar)	Duration (min)
1	DuoPAP	0	15	4
2	DuoPAP	5	20	4
3	DuoPAP	7	22	4
4	DuoPAP	10	25	4
5	DuoPAP	15	30	4
6	DuoPAP	20	35	4
7	DuoPAP	0	15	4

Respiratory rate was maintained at 15 breaths/min, inspiratory time at 1.35 s, and flow-trigger sensitivity at 10 L/min. Each ventilation condition was recorded for 4 minutes and repeated three times.

### 2.6 Technical feasibility and repeatability assessment

#### 2.6.1 Chamber-pressure stability

Chamber pressure was continuously recorded during all measurements. The negative-pressure chamber was operated at a fixed vacuum setting of approximately −7 mbar, and this vacuum level was not actively regulated during mechanical ventilation. Before each ventilation sequence, gross lung expansion was visually inspected to exclude obvious macroscopic collapse or major asymmetrical inflation. This visual assessment was not interpreted as evidence of complete alveolar recruitment. Although the target vacuum remained constant, the measured chamber-pressure signal exhibited cycle-dependent fluctuations, with expiratory pressures remaining close to the negative baseline at −7.482 ± 0.232 mbar and brief positive excursions reaching up to +1.821 ± 0.303 mbar during inspiration. These transient positive pressures are not measurement artifacts and do not indicate a failure of the ventilation setup. They arise from the passive mechanical coupling between lung expansion and the fixed-volume chamber: as the lung inflates, the expanding lung displaces volume within the sealed chamber, causing the chamber pressure to rise and, in some cases, exceed atmospheric pressure. Importantly, the chamber-pressure setting itself remained negative throughout the experiment; only the instantaneous measured pressure exhibited short positive deviations due to volume displacement.

For each ventilation cycle and repetition, chamber-pressure stability and phase-dependent variation were quantified using median pressure, standard deviation, minimum, maximum, and peak-to-peak range. Chamber pressure represents a defined extrathoracic boundary condition of the simulator and does not correspond to, or reproduce, a physiological intrapleural pressure waveform.

#### 2.6.2 Within-specimen technical repeatability

Each PEEP condition was measured three times using the same lung specimen. For each repetition, regional pressure metrics, tidal volume, dynamic compliance, and chamber-pressure characteristics were calculated. Condition-specific values were summarized across the three repeated measurements, and short-term technical repeatability was characterized using the observed dispersion across repetitions. Because all repetitions were obtained from the same specimen, this analysis represents technical within-specimen repeatability and does not assess biological reproducibility or inter-specimen variability.

### 2.7 Signal processing

Signal processing was performed using custom Python scripts based on NumPy, Pandas, and Matplotlib (Figure 3). TDMS pressure values were converted according to

$$P_{r}\left[n\right]=\frac{{Raw}_{r}\left[n\right]}{100}$$ (1a)

and the TDMS time axis was reconstructed using the known sampling frequency of 100 Hz (Equation 1a).

For regional pressure analysis, tracheal pressure P_tr_(t) (Equation 1) served as the proximal reference. Regional pressure attenuation was defined as

$$\Delta P_{r}\left(t\right)=P_{tr}\left(t\right)-P_{r}\left(t\right)$$ (1)

where P_r_(t) denotes pressure measured at regional site *r*.

No digital low-pass or high-pass filtering was applied to the regional pressure signals used for quantitative pressure analysis. A short moving-average filter was used only for signal synchronization and automated respiratory-phase detection, as described below.

#### 2.7.1 Time synchronization

Because regional pressure signals and FlowAnalyser signals were acquired by independent recording systems, temporal synchronization was performed using cross-correlation. The first derivative of the tracheal pressure signal was calculated numerically and smoothed using a 50-ms moving-average filter. The FlowAnalyser airflow signal was interpolated onto the common 100-Hz time grid and smoothed using the same moving-average window. Both signals were z-normalized before cross-correlation.

To account for a possible polarity mismatch between recording systems, cross-correlation was evaluated for both the original airflow signal Q(t) and its inverted form −Q(t). The polarity yielding the larger correlation was retained. The temporal lag maximizing the cross-correlation was determined within a predefined window of ±10 s, and the FlowAnalyser time axis was shifted by the resulting lag. All subsequent pressure-flow and volume analyses were performed using the synchronized signals.

#### 2.7.2 Respiratory phase detection

Respiratory phase transitions were identified from the first derivative of the tracheal pressure signal P_tr_(t). The derivative was calculated numerically at 100 Hz and smoothed using a 50-ms moving-average filter. A data-adaptive detection threshold was defined as 15% of the 95th percentile of the absolute smoothed pressure derivative.

Positive threshold crossings identified the onset of inspiration, whereas negative threshold crossings identified the onset of expiration. To reduce repeated or spurious detections, a hysteresis threshold corresponding to 50% of the primary detection threshold, a minimum interval of 300 ms between consecutive transition events, and a minimum threshold persistence of 80 ms were applied. Inspiratory and expiratory phases were defined as the intervals between consecutive positive and negative transitions, respectively.

For standardized batch analysis, a maximum analysis window of 170 s beginning with the first detected inspiration was used. The detected respiratory segments were subsequently applied to all phase-specific pressure, pressure-gradient, pressure-flow, tidal-volume, and compliance analyses.

#### 2.7.3 Regional pressure-flow index

For each regional measurement site, pressure differences relative to tracheal pressure were analyzed separately during inspiration and expiration. An effective regional pressure-flow (Equation 2) index was calculated as

$$R_{r}\left(t\right)=\frac{{\Delta P}_{r}\left(t\right)}{Q\left(t\right)}=\frac{P_{tr}\left(t\right)-P_{r}\left(t\right)}{Q\left(t\right)}$$ (2)

where P_tr_(t) is the tracheal pressure, P_r_(t) is the pressure measured at regional site r, and Q(t) is the synchronized airflow measured by the FlowAnalyser. Before pressure-flow analysis, the airflow signal was corrected for a constant baseline offset by subtracting its mean over the analyzed interval. Samples with zero or near-zero flow were excluded from the calculation to avoid numerical instability.

R_r_(t) was interpreted as an effective pressure-flow or impedance surrogate rather than anatomically isolated airway resistance, because the measured pressure difference includes contributions from the endotracheal tube, connector geometry, regional airway pathways, and tissue mechanics.

#### 2.7.4 Tidal-volume reconstruction and dynamic compliance

Tidal volume (TV) was reconstructed from the synchronized airflow signal. Following baseline-offset correction, airflow was converted from L/min to L/s. Cumulative volume was calculated by trapezoidal numerical integration using a sampling interval of 0.01s.

To compensate for residual cumulative integration drift, the integrated volume values at successive inspiratory onsets were used as anchor points. A first-order linear trend was fitted to these anchor values over time and subtracted from the cumulative volume signal. Tidal volume for each respiratory cycle was then calculated as the difference in drift-corrected volume between the beginning and end of inspiration.

Dynamic system compliance (Equation 3) was calculated for each inspiratory cycle as

$$C_{dyn,k}=\Delta V_{k}/P_{tr,end,k}-P_{tr,start,k}$$ (3)

where ΔV_k_ represents the reconstructed tidal-volume change during inspiration and P_tr,start,k_ and P_tr,end,k_ denote tracheal pressure at the beginning and end of the corresponding inspiration. Dynamic compliance was interpreted as a system-level metric of the complete ex-vivo lung, airway interface, endotracheal tube, and chamber configuration rather than as isolated parenchymal lung compliance.

#### 2.7.5 Trachea-to-region pressure-transmission regression

Pressure transmission from the trachea to each regional measurement site was characterized using linear regression of regional pressure against tracheal pressure. The regression slope, intercept, Pearson correlation coefficient r, coefficient of determination R^2^, and standard deviation of the residuals were extracted as descriptive measures of trachea-to-region pressure coupling.

For hysteresis analysis, inspiratory and expiratory regional pressure trajectories were evaluated separately as functions of tracheal pressure. To reduce scatter, regional pressure values were summarized in tracheal-pressure bins using median values. Inspiratory and expiratory trajectories were subsequently min-max normalized (Equation 4) independently to the interval [0,1]:

$$x_{norm}=\frac{x-x_{\min}}{x_{\max}-x_{\min}},y_{norm}=\frac{y-y_{\min}}{y_{\max}-y_{\min}}$$ (4)

The normalized inspiratory and expiratory branches were interpolated onto a common tracheal-pressure grid of 300 points. A dimensionless pressure-trajectory hysteresis area (Equation 5) was calculated by trapezoidal integration of the absolute distance between both branches:

$$A_{hyst}=\int_{x_{\min}}^{x_{\max}}\left|y_{insp}\left(x\right)-y_{\exp}\left(x\right)\right|dx$$ (5)

For visualization and inter-condition comparison, hysteresis areas were additionally normalized (Equation 6) to the largest hysteresis area observed across all region–measurement combinations:

$$A_{hyst,norm}=\frac{A_{hyst}}{A_{hyst,\max}}$$ (6)

Because the metric is derived from normalized pressure-pressure trajectories rather than a pressure-volume loop, it was interpreted as a dimensionless index of phase-dependent pressure-transmission hysteresis and not as mechanical work or energy dissipation.

#### 2.7.6 Poincaré dispersion analysis

Poincaré analysis was used as an exploratory descriptor of regional pressure-state dispersion. For each measurement and anatomical region, the regional pressure values were ordered according to the corresponding tracheal-pressure sequence and successive pairs x_n_ and x_n+1_ were represented in two-dimensional state space.

The dispersion perpendicular and parallel to the identity line was quantified using SD1 and SD2 (Equation 7), respectively:

$$SD1=SD\;\left(\frac{x_{n+1}-x_{n}}{\sqrt{2}}\right)$$ (7)

and their ratio was calculated as

$$\Delta SD=\frac{SD1}{SD2}$$ (8)

ΔSD (Equation 8) was interpreted as a dimensionless descriptor of the relative short-axis to long-axis dispersion of the pressure-state distribution. Because the data sequence used for this analysis was not treated as an uninterrupted temporal series, SD1 and SD2 were not interpreted as conventional short-term and long-term temporal variability measures.

For visualization of PEEP-dependent changes, ΔSD values were normalized separately within each anatomical region to the maximum ΔSD observed for that region across the investigated PEEP conditions.

#### 2.7.7 Cycle normalization, visualization, and descriptive analysis

Respiratory cycles were detected automatically using the phase-detection procedure described above. For visualization of mean inspiratory and expiratory trajectories, individual respiratory phases were interpolated to the median phase duration before averaging. Mean trajectories and corresponding standard deviations were calculated across detected cycles.

Regional pressure metrics were additionally mapped to anatomical lung regions and visualized using regional heatmaps. Because the study used a single biological specimen and repeated respiratory cycles do not represent independent biological observations, statistical analyses were descriptive. Pressure and pressure-difference distributions were summarized using medians, quartiles, ranges, and standard deviations as appropriate, whereas tidal volume and dynamic compliance were summarized across detected cycles and repeated measurements. No inferential hypothesis testing regarding biological differences between lung regions or PEEP conditions was performed, and no multiple-comparison procedure was applied.

### 2.8 Statistical analysis

Because this study was designed as a technical feasibility and system-characterization investigation using a single lung specimen, statistical analyses were primarily descriptive. Regional pressure measurements, pressure differences, pressure-flow indices, tidal-volume estimates, dynamic compliance, pressure-trajectory hysteresis, and Poincaré-derived dispersion metrics were summarized using medians, interquartile ranges, ranges, and standard deviations, as appropriate. Repeated respiratory cycles and repeated ventilation sequences were considered technical repeated observations within the same specimen and were not treated as independent biological replicates. Accordingly, no inferential statistical testing of differences between anatomical regions or PEEP conditions was performed, and no adjustment for multiple comparisons was required.

### 2.9 Ethical considerations

The porcine lungs used in this study were commercially available anatomical specimens. According to the manufacturer, the tissue originates from animals processed within the regular food production chain and preserved for educational or technical use.

No animals were sacrificed or subjected to experimental procedures for this study. The experiments therefore do not constitute animal experimentation according to European Directive 2010/63/EU or the German Animal Welfare Act.

The animal welfare officer of the Justus Liebig University Giessen, Germany confirmed that no further ethical approval was required.

## 3 Results

### 3.1 System operation and synchronized signal acquisition

The integrated measurement system enabled simultaneous acquisition of seven regional intrapulmonary pressure channels, airway pressure, airflow, and chamber pressure across all ventilation conditions. TDMS pressure recordings and FlowAnalyser data were synchronized using the cross-correlation-based procedure described in the Methods and interpolated onto a common time base. Automated respiratory-phase detection provided reproducible inspiratory and expiratory segments for subsequent phase-specific analyses.

### 3.2 Chamber-pressure stability and global ventilation-derived metrics

The vacuum system was operated with a nominal chamber-pressure setting of approximately −7 mbar throughout all ventilation conditions. Across the three repeated measurements per condition, the chamber-pressure summary values remained consistently subatmospheric, ranging from −7.165 to −7.806 mbar across the investigated PEEP levels (Table 2).

**TABLE 2:** Technical within-specimen repeatability, chamber-pressure summary values, reconstructed tidal volume, and dynamic system compliance across PEEP conditions. Each ventilation condition was repeated three times.

PEEP condition	Repetitions	Chamber-pressure summary (mbar)	VT median	Cdyn median
0 mbar	3	−7.539 mbar (±1.550 mbar)	263.5 mL (±2.8 mL)	20.3 ± 0.4 mL/mbar
5 mbar	3	−7.806 mbar (±1.604 mbar)	278.1 mL (±1.3 mL)	20.1 ± 0.1 mL/mbar
7 mbar	3	−7.689 mbar (±1.818 mbar)	282.7 mL (±1.0 mL)	20.5 ± 0.2 mL/mbar
10 mbar	3	−7.493 mbar (±1.864 mbar)	280.9 mL (±1.2 mL)	20.1 ± 0.2 mL/mbar
15 mbar	3	−7.165 mbar (±1.940 mbar)	277.8 mL (±1.4 mL)	19.4 ± 0.1 mL/mbar
20 mbar	3	−7.285 mbar (±1.992 mbar)	270.6 mL (±1.2 mL)	19.5 ± 0.1 mL/mbar
0 after PEEP 20	3	−7.565 mbar (±1.497 mbar)	294.1 mL (±1.5 mL)	21.2 ± 0.3 mL/mbar

Superimposed on this stable subatmospheric baseline, the chamber-pressure signal exhibited respiratory-cycle-dependent fluctuations. During expiration, chamber pressure remained close to the negative baseline (−7.482 ± 0.232 mbar), whereas lung expansion during mechanical inspiration caused brief pressure excursions that reached up to +1.822 ± 0.303 mbar. Thus, the positive values represent transient inspiratory excursions of the instantaneous chamber-pressure signal and not a change of the chamber-pressure operating point or the condition-specific central pressure reported in Table 2.

`These transient excursions are attributed to passive volume displacement within the sealed chamber as the lung expands during positive-pressure inspiration. Accordingly, the chamber should be regarded as providing a reproducible subatmospheric baseline boundary condition with superimposed cycle-dependent pressure fluctuations, rather than as reproducing a continuously negative physiological pleural-pressure waveform. Tidal volume and dynamic system compliance were reconstructed from the synchronized flow and pressure signals and are summarized together with chamber-pressure stability in Table 2.

### 3.3 Regional distribution of intrapulmonary pressures dynamics

Regional intrapulmonary pressures were recorded simultaneously at seven anatomical locations within the lung model, including the trachea, the right and left main bronchi, and the upper and lower lobes of both lungs. Measurements were acquired during pressure-controlled ventilation across PEEP levels from 0 to 20 mbar.

Repeated measurements showed high short-term within-specimen repeatability. Across the three repeated measurements per PEEP level, median regional pressure values differed by approximately ≈0.02 mbar, indicating stable pressure acquisition and consistent signal processing under identical experimental conditions.

Across all measurement sites, both inspiratory and expiratory pressures increased progressively with rising PEEP. At PEEP 0, inspiratory pressures ranged between approximately 14 and 15 mbar across the lung regions, while expiratory pressures remained close to atmospheric pressure. With increasing PEEP, inspiratory pressures rose steadily and reached approximately 32–33 mbar at PEEP 20. Expiratory pressures increased proportionally with the applied PEEP and closely followed the ventilator setpoints.

Within the investigated specimen, the spatial distribution of absolute intrapulmonary pressure remained largely homogeneous despite the increase in absolute pressure with PEEP. Differences between bronchial and lobar measurement sites were small and typically remained below approximately 1–2 mbar. Tracheal pressure closely matched the ventilator waveform, confirming stable mechanical coupling between the ventilator circuit and the lung model.

The inspiratory–expiratory pressure difference, corresponding to the applied driving pressure, remained relatively constant across the investigated PEEP levels. Across all regions, ΔP typically ranged between approximately 12 and 15 mbar.

Minor regional deviations were observed under low PEEP conditions. At PEEP 0, ΔP values varied by up to approximately 3–4 mbar between regions, with the left lower lobe exhibiting the highest values. Within this specimen, regional differences became smaller at higher PEEP levels, resulting in a more uniform measured pressure distribution.

Anatomical heatmaps (Figure 4) illustrate median inspiratory pressures, expiratory pressures, and the inspiratory–expiratory pressure difference ΔP across the lung regions. Bar charts summarize the corresponding pressure values for each anatomical location.

**FIGURE 4:**
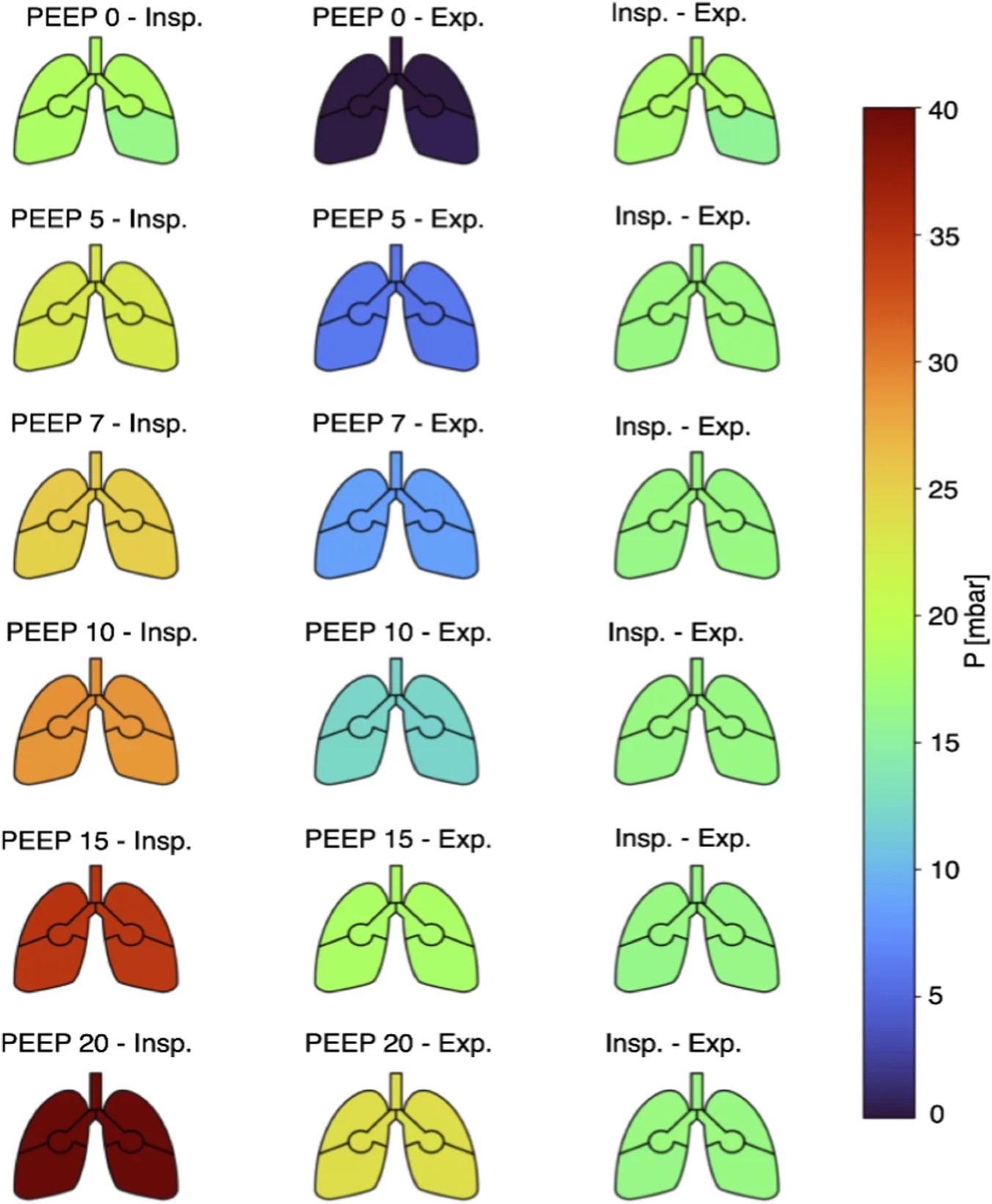
Regional intrapulmonary absolute pressure distribution across lung regions during ventilation at increasing PEEP levels.

### 3.4 Pressure transmission relative to tracheal pressure

Figure 4 was intentionally retained as an overview of the absolute regional inspiratory and expiratory pressure distribution across PEEP conditions. This representation complements the trachea-referenced analysis presented in the present section, which specifically quantifies regional pressure transmission relative to the proximal tracheal reference. While absolute regional pressures largely followed the ventilator pressure levels, the trachea-to-region analysis revealed small but measurable regional deviations, particularly in the left lower lung lobe at PEEP 0.

To assess how airway pressure propagates from the trachea into the lung periphery, regional pressure differences relative to tracheal pressure were calculated.

Across most regions and ventilation conditions, these gradients remained small and typically below 1 mbar. Bronchial measurement sites showed the smallest deviations from tracheal pressure, reflecting their direct anatomical connection to the central airways.

Peripheral regions showed slightly larger deviations at low PEEP levels. The largest gradient was observed in the left lower lobe at PEEP 0, where inspiratory pressure exceeded tracheal pressure by approximately 3.2 mbar, while expiratory pressure was about 1.0 mbar lower than tracheal pressure.

With increasing PEEP, regional pressure gradients progressively decreased and became more uniform across the lung model, resulting in smaller measured trachea-to-region pressure differences within the investigated specimen.

Heatmaps show inspiratory and expiratory pressure gradients relative to tracheal pressure. Bar charts provide quantitative comparisons across anatomical regions.

### 3.5 Regional hysteresis during the respiratory cycle

To investigate regional mechanical behavior during ventilation, hysteresis loops were calculated between tracheal pressure and regional intrapulmonary pressures. The area between normalized inspiratory and expiratory pressure trajectories was used as a dimensionless index of phase-dependent pressure-transmission hysteresis.

Clear regional differences were observed between central airway regions and peripheral lung regions. Bronchial measurement sites showed nearly overlapping inspiratory and expiratory trajectories across all PEEP levels, resulting in minimal hysteresis areas (Figure 5).

**FIGURE 5:**
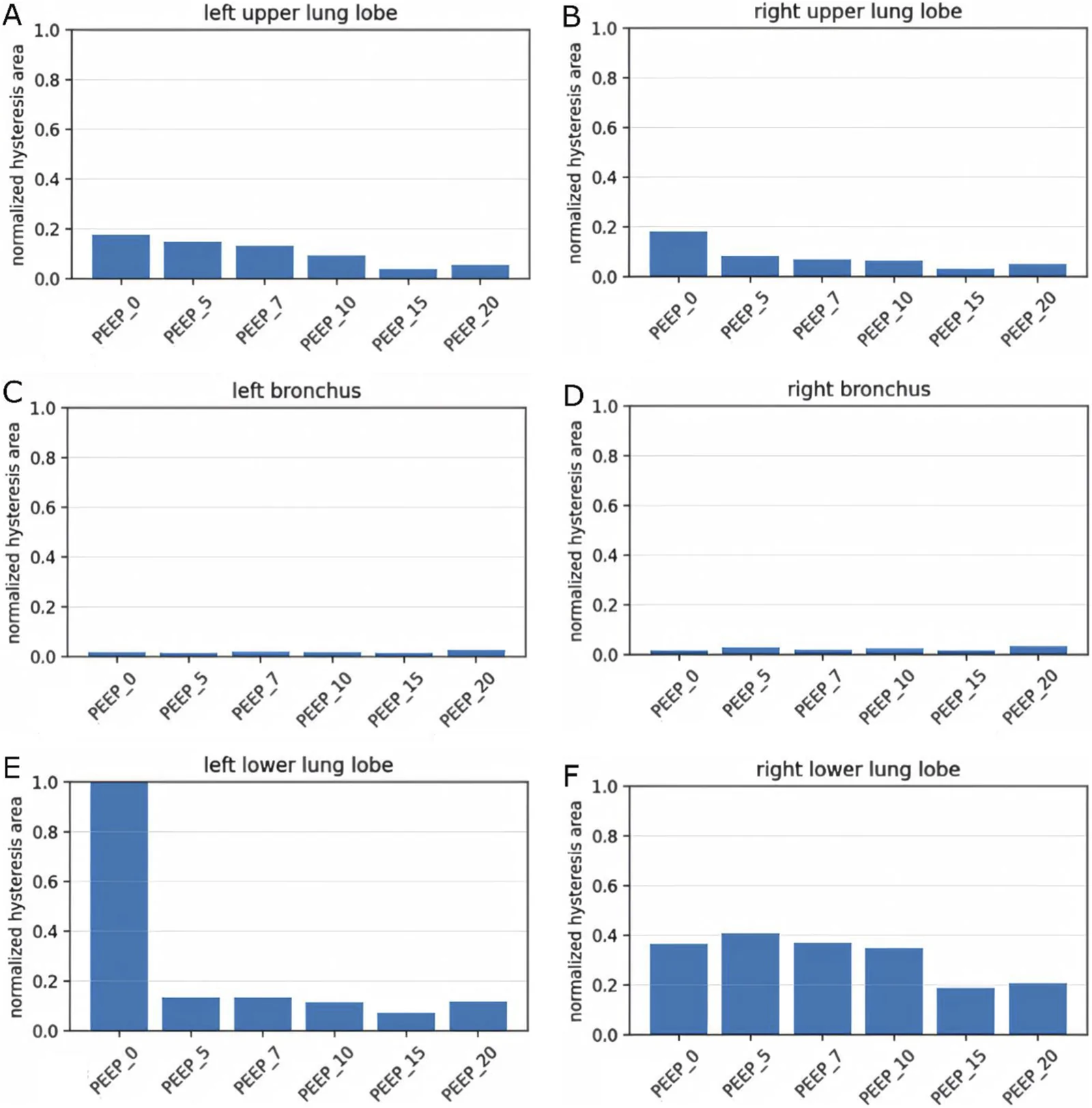
Each panel shows the hysteresis area normalized to the respective maximum value for the PEEP levels of 0, 5, 7, 10, 15, and 20 cmH_2_O. **(A)** left upper lung lobe **(B)** right upper lung lobe **(C)** left bronchus **(D)** right bronchus **(E)** left lower lung lobe **(F)** right lower lung lobe. In the lung lobes **(A,B,E,F)**, hysteresis tends to decrease as PEEP increases, while in both bronchi **(C,D)**, it remains consistently minimal across all pressure levels.

In contrast, peripheral lung regions demonstrated distinct hysteresis loops with measurable separation between inspiratory and expiratory trajectories (Figure 6).

**FIGURE 6:**
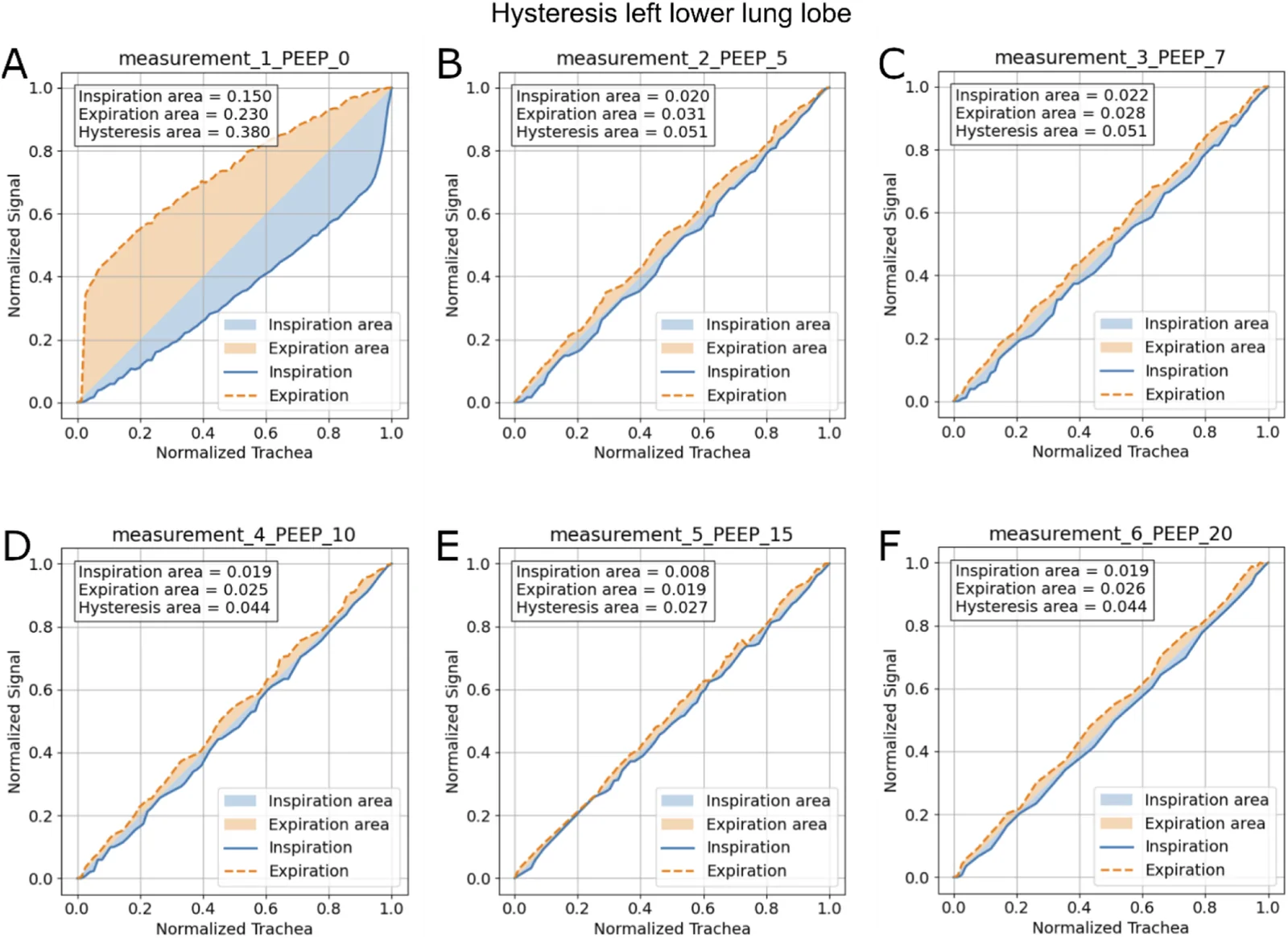
Hysteresis loops of the left lower lung lobe across PEEP levels. Each panel shows the normalized regional pressure signal plotted against the normalized tracheal pressure during inspiration (blue, solid) and expiration (orange, dashed), with the inspiration area (blue) and expiration area (orange) shaded and the resulting hysteresis area indicated. **(A)** Measurement 1, PEEP 0 (hysteresis area = 0.380). **(B)** Measurement 2, PEEP 5 (hysteresis area = 0.051). **(C)** Measurement 3, PEEP 7 (hysteresis area = 0.051). **(D)** Measurement 4, PEEP 10 (hysteresis area = 0.044). **(E)** Measurement 5, PEEP 15 (hysteresis area = 0.027). **(F)** Measurement 6, PEEP 20 (hysteresis area = 0.044). The hysteresis area is larger at PEEP 0 **(A)** than at higher PEEP levels **(B–F)**.

Bronchial regions show minimal hysteresis, whereas lobar regions demonstrate larger hysteresis areas, particularly at low PEEP.

#### 3.5.1 Lower lobes

The most pronounced hysteresis behavior was observed in the lower lobes. In the right lower lobe, inspiratory and expiratory trajectories remained separated across ventilation conditions, with the largest hysteresis areas observed at lower PEEP levels. An even more pronounced effect was observed in the left lower lobe (Figure 5). At PEEP 0, this region showed the largest normalized pressure-trajectory hysteresis area observed in the study. Figure 6 illustrates the trajectory-level basis of this finding: at PEEP 0, the inspiratory and expiratory branches were markedly separated, resulting in a hysteresis area of 0.380. At PEEP 5, the area decreased to 0.051 and remained comparatively low across the subsequent PEEP conditions. Thus, the prominent value in Figure 5 reflects a markedly broader phase-dependent pressure-transmission loop at PEEP 0 rather than a generalized increase in hysteresis across all ventilation conditions. With increasing PEEP, hysteresis areas decreased substantially.

#### 3.5.2 Upper lobes

Upper lobar regions showed moderate hysteresis behavior.

In the left upper lobe, inspiratory and expiratory trajectories diverged slightly at low PEEP levels but became progressively aligned as PEEP increased.

#### 3.5.3 Bronchial regions

In contrast to lobar regions, bronchial measurement sites exhibited minimal hysteresis loops. Both the right bronchus and left bronchus showed nearly overlapping inspiratory and expiratory trajectories across all ventilation conditions.

#### 3.5.4 Poincaré-derived regional pressure dispersion

Poincaré analysis was used to characterize regional pressure-state dispersion using the dimensionless ratio ΔSD = SD1/SD2. Broader pressure-state distributions were observed in peripheral regions, particularly under low-PEEP conditions in the investigated specimen. With increasing PEEP, several peripheral regions showed narrower Poincaré distributions and lower relative dispersion. For visualization of PEEP-dependent within-region changes, ΔSD was normalized separately to the maximum value observed within each anatomical region (Figure 7).

**FIGURE 7:**
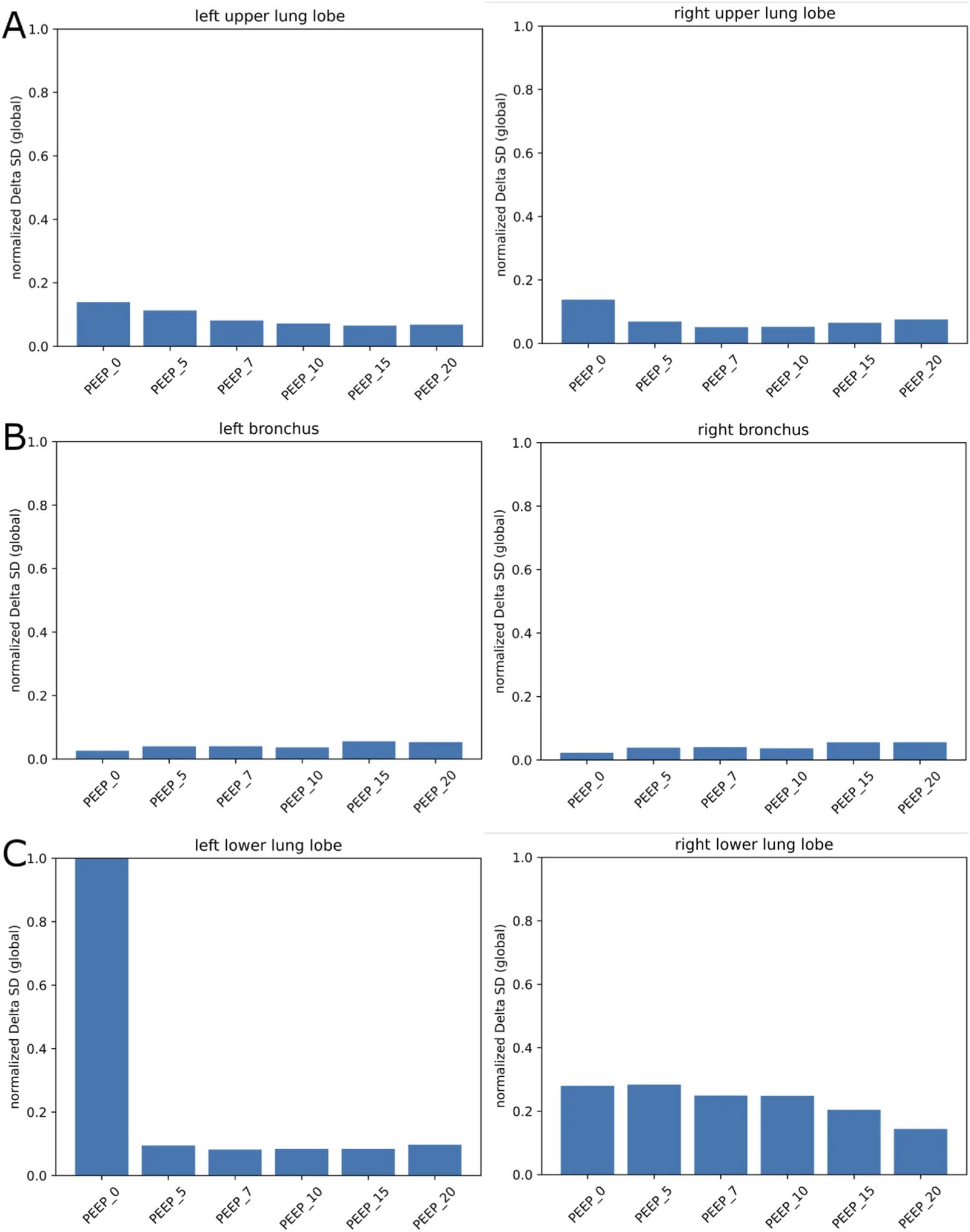
Normalized global ΔSD across lung regions and PEEP levels. Each panel shows the ΔSD (global), normalized to the respective maximum value, for the PEEP levels 0, 5, 7, 10, 15, and 20 cmH_2_O. **(A)** Left and right upper lung lobe. **(B)** Left and right bronchus. **(C)** Left and right lower lung lobe. In the upper and lower lung lobes **(A,C)**, normalized ΔSD tends to decrease as PEEP increases, in the left lower lung lobe, while in both bronchi **(B)** it remains consistently minimal across all pressure levels.

Figures 8–11 illustrate the underlying Poincaré distributions of the peripheral measurement regions. The most pronounced regional pattern was observed in the left lower lung lobe at PEEP 0 (Figure 9). Under this condition, SD1 was 2.612 mbar and SD2 was 4.352 mbar, resulting in a ΔSD of 0.600. In contrast, ΔSD ranged only between 0.049 and 0.059 at PEEP levels from 5 to 20 mbar. The comparatively broad distribution at PEEP 0 therefore reflects a substantially greater component of pressure-state dispersion perpendicular to the identity line relative to the dominant along-axis dispersion. With increasing PEEP, the distribution became markedly narrower and more closely aligned with the identity line, indicating a more constrained regional pressure-state relationship within this specimen. A less pronounced reduction in relative dispersion was also observed in the right lower lung lobe, whereas the upper lobes showed substantially smaller changes.

**FIGURE 8:**
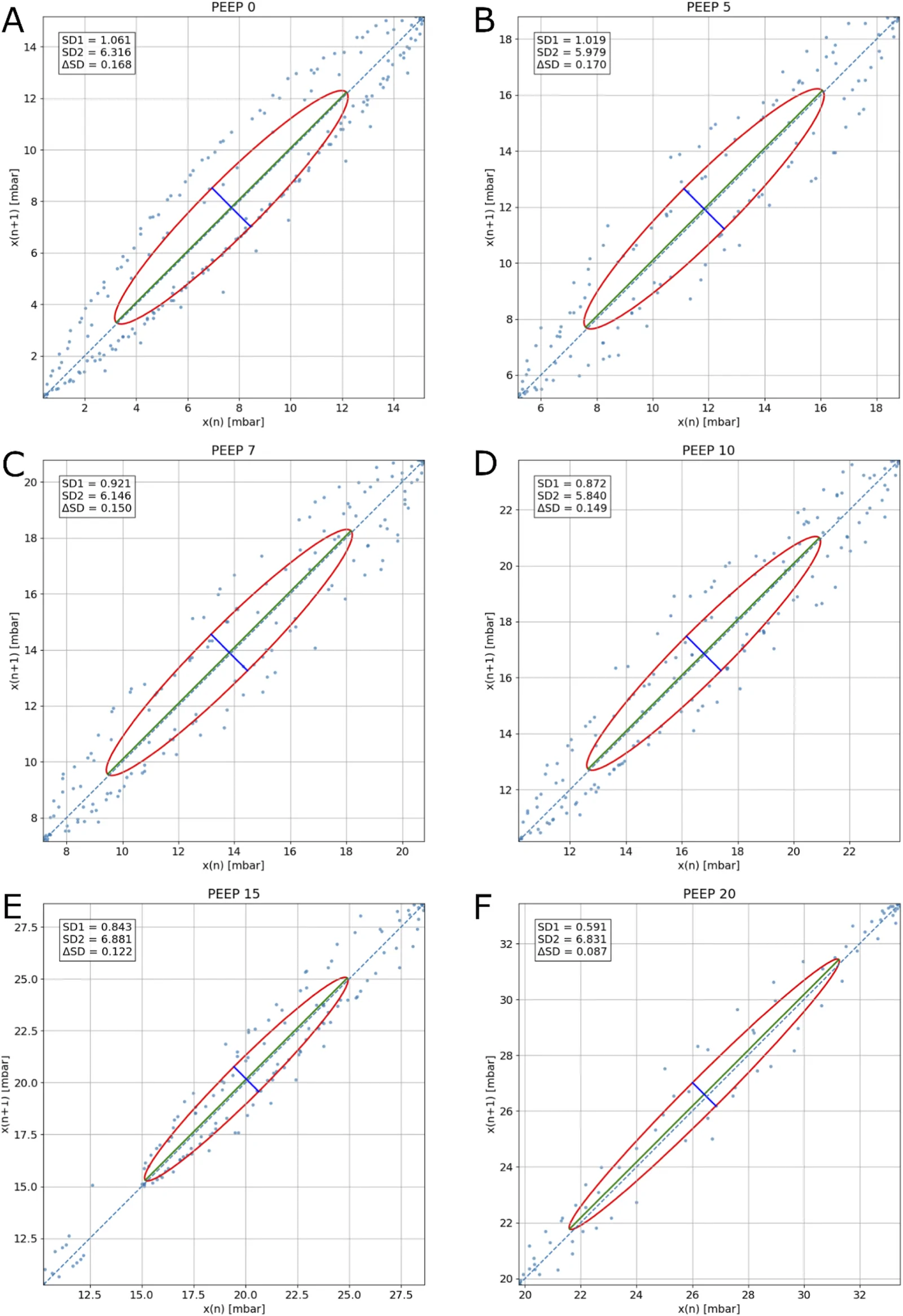
Poincaré plots for peripheral lung region (right lower lung lobe). Each panel shows a Poincaré plot of x(n) versus x (n+1) mbar. The fitted ellipse illustrates dispersion perpendicular to the identity line (SD1) and parallel to the identity line (SD2), respectively. **(A)** PEEP 0 (SD1 = 1.061, SD2 = 6.316, ΔSD = 0.168). **(B)** PEEP 5 (SD1 = 1.019, SD2 = 5.979, ΔSD = 0.170). **(C)** PEEP 7 (SD1 = 0.921, SD2 = 6.146, ΔSD = 0.150). **(D)** PEEP 10 (SD1 = 0.872, SD2 = 5.840, ΔSD = 0.149). **(E)** PEEP 15 (SD1 = 0.843, SD2 = 6.881, ΔSD = 0.122). **(F)** PEEP 20 (SD1 = 0.591, SD2 = 6.831, ΔSD = 0.087).

**FIGURE 9:**
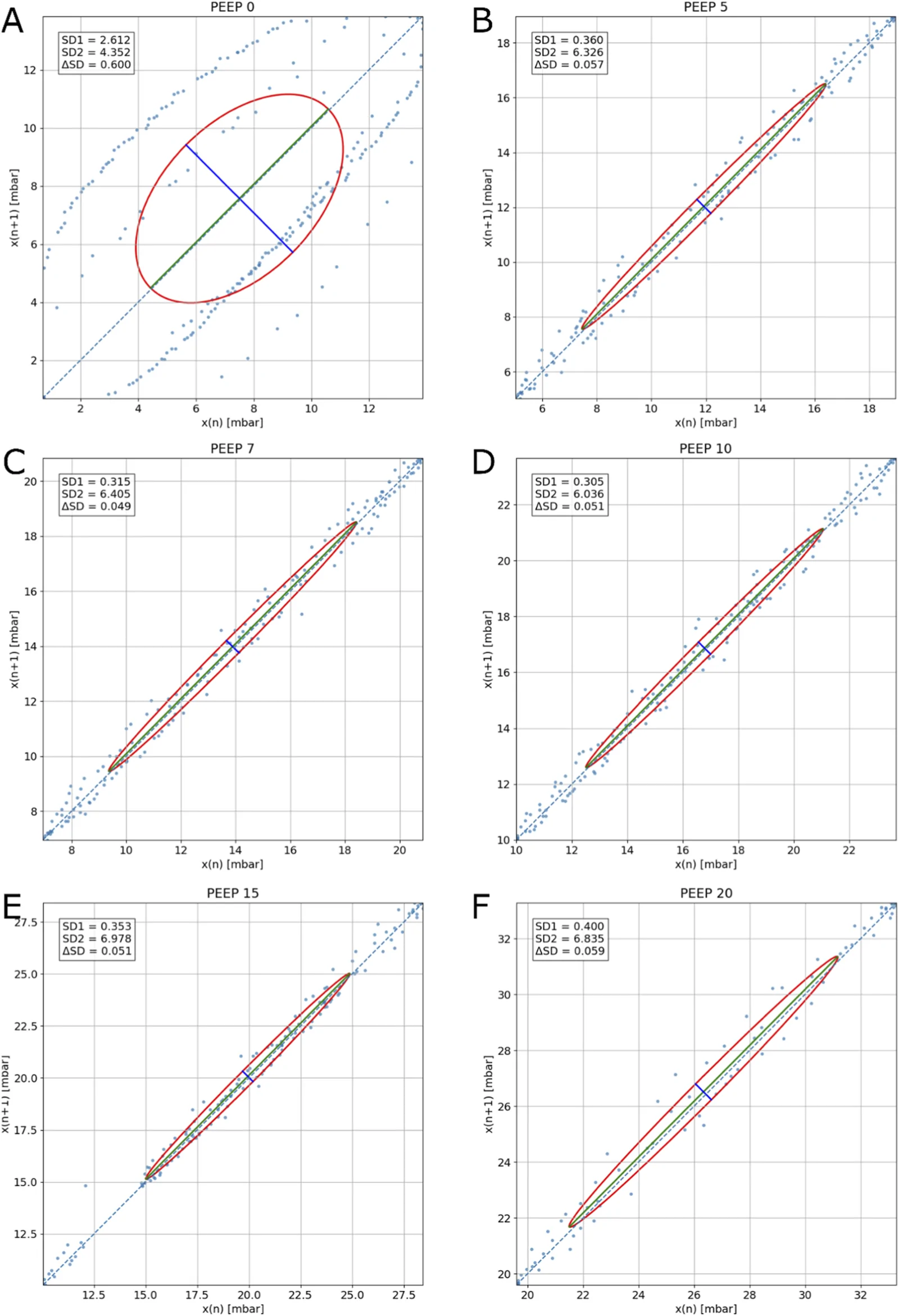
Poincaré plots for peripheral lung region (left lower lung lobe). Each panel shows a Poincaré plot of x(n) versus x (n+1) mbar. The fitted ellipse illustrates dispersion perpendicular to the identity line (SD1) and parallel to the identity line (SD2), respectively. **(A)** PEEP 0 (SD1 = 2.612, SD2 = 4.352, ΔSD = 0.600). **(B)** PEEP 5 (SD1 = 0.360, SD2 = 6.326, ΔSD = 0.057). **(C)** PEEP 7 (SD1 = 0.315, SD2 = 6.405, ΔSD = 0.049). **(D)** PEEP 10 (SD1 = 0.305, SD2 = 6.036, ΔSD = 0.051). **(E)** PEEP 15 (SD1 = 0.353, SD2 = 6.978, ΔSD = 0.051). **(F)** PEEP 20 (SD1 = 0.400, SD2 = 6.835, ΔSD = 0.059).

**FIGURE 10:**
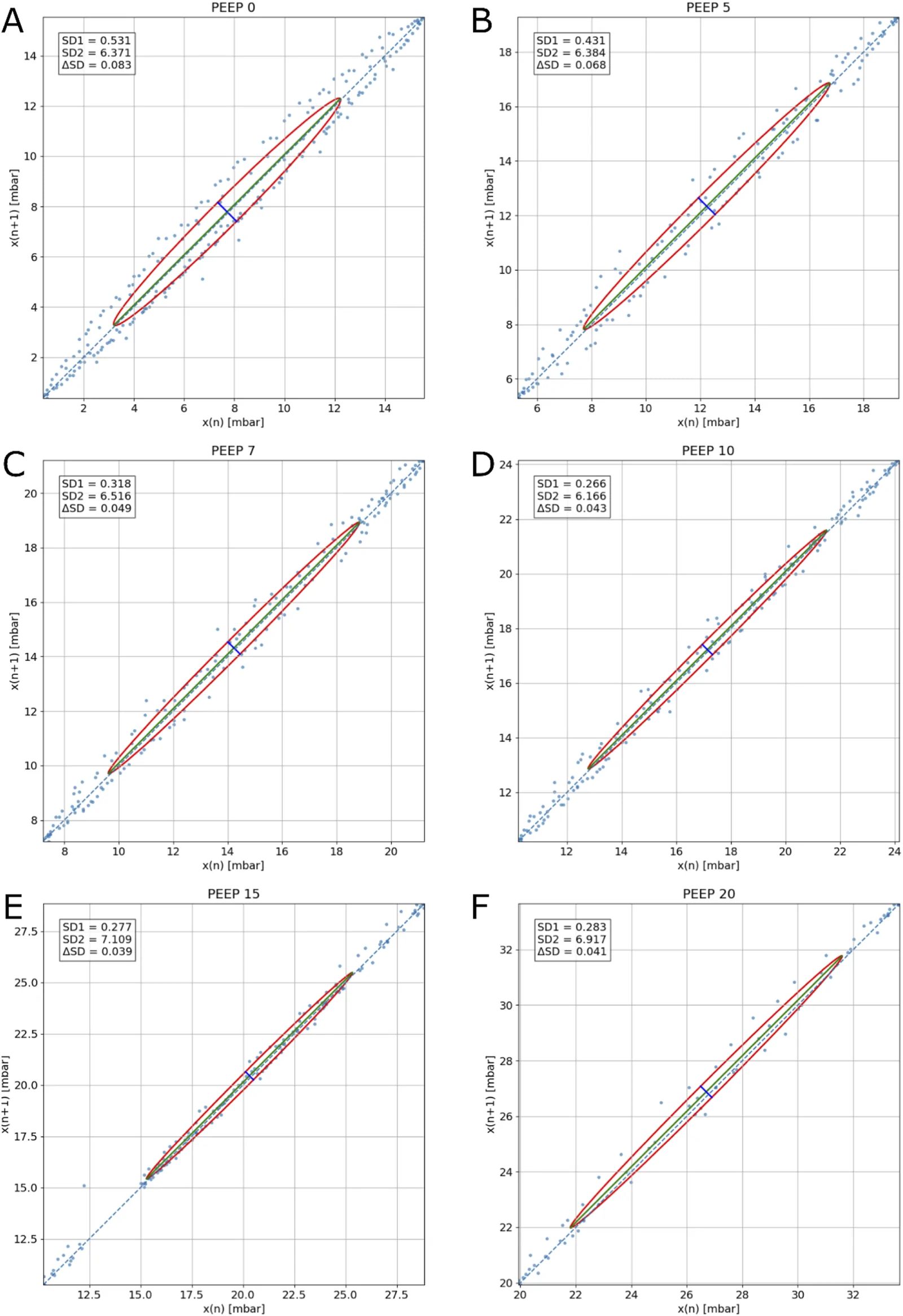
Poincaré plots for peripheral lung region (left upper lung lobe). Each panel shows a Poincaré plot of x(n) versus x (n+1) mbar. The fitted ellipse illustrates dispersion perpendicular to the identity line (SD1) and parallel to the identity line (SD2), respectively. **(A)** PEEP 0 (SD1 = 0.531, SD2 = 6.371, ΔSD = 0.083). **(B)** PEEP 5 (SD1 = 0.431, SD2 = 6.384, ΔSD = 0.068). **(C)** PEEP 7 (SD1 = 0.318, SD2 = 6.516, ΔSD = 0.049). **(D)** PEEP 10 (SD1 = 0.266, SD2 = 6.166, ΔSD = 0.043). **(E)** PEEP 15 (SD1 = 0.277, SD2 = 7.109, ΔSD = 0.039). **(F)** PEEP 20 (SD1 = 0.283, SD2 = 6.917, ΔSD = 0.041).

**FIGURE 11:**
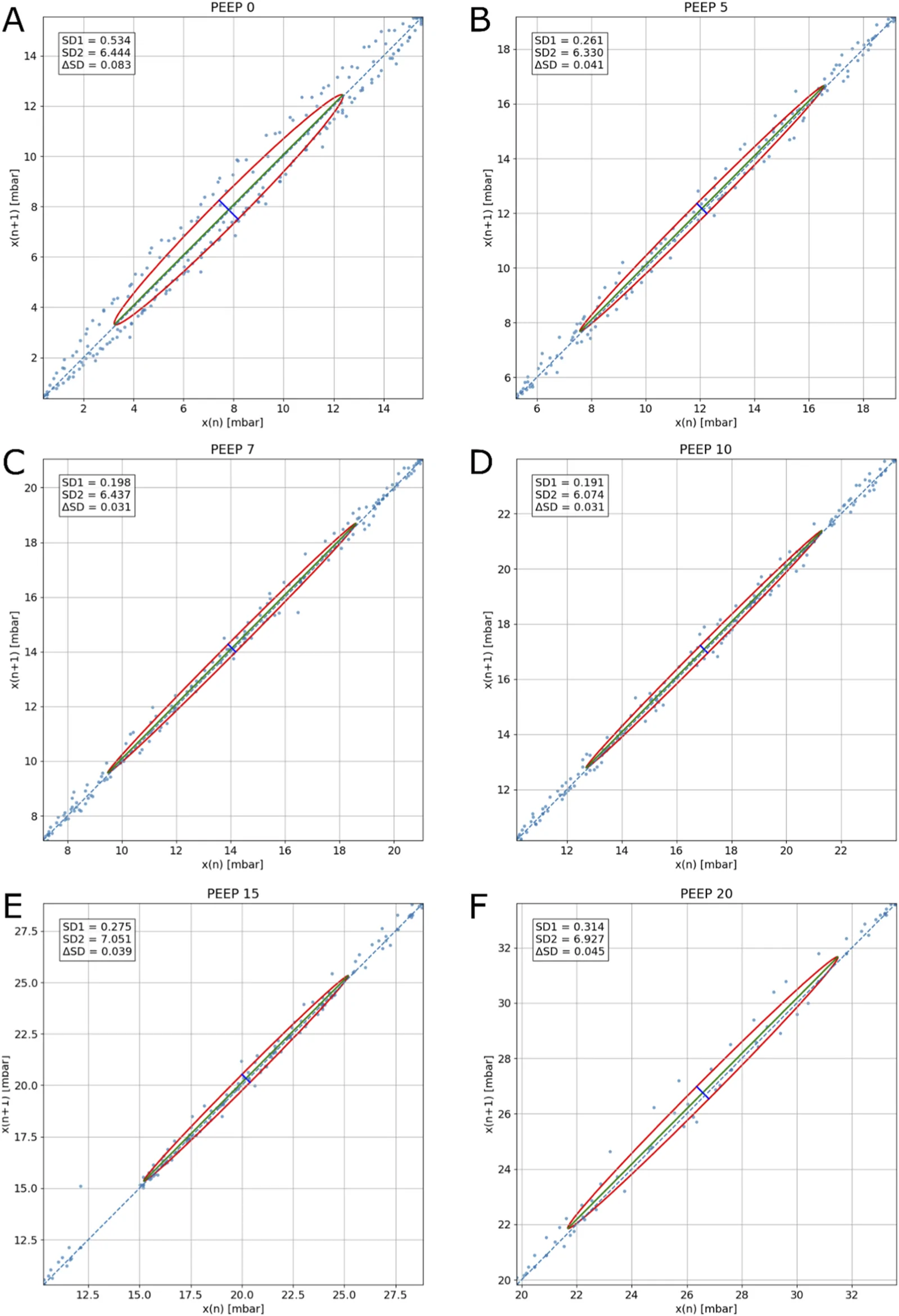
Poincaré plots for peripheral lung region (right upper lung lobe). Each panel shows a Poincaré plot of x(n) versus x (n+1) mbar. The fitted ellipse illustrates dispersion perpendicular to the identity line (SD1) and parallel to the identity line (SD2), respectively. **(A)** PEEP 0 (SD1 = 0.534, SD2 = 6.444, ΔSD = 0.083). **(B)** PEEP 5 (SD1 = 0.261, SD2 = 6.330, ΔSD = 0.041). **(C)** PEEP 7 (SD1 = 0.198, SD2 = 6.437, ΔSD = 0.031). **(D)** PEEP 10 (SD1 = 0.191, SD2 = 6.074, ΔSD = 0.031). **(E)** PEEP 15 (SD1 = 0.275, SD2 = 7.051, ΔSD = 0.039). **(F)** PEEP 20 (SD1 = 0.314, SD2 = 6.927, ΔSD = 0.045).

Representative Poincaré plots for peripheral lung regions and upper lobes are shown in Figures 8–11, whereas bronchial regions show compact distributions with minimal variability.

#### 3.5.5 Effect of prior high-PEEP exposure on regional pressure hysteresis

In the investigated specimen, peripheral regions showed lower pressure-trajectory hysteresis after prior exposure to PEEP 20 mbar. The largest change was observed in the left lower lung region, where inspiratory and expiratory trajectories became substantially more aligned (Figure 12).

**FIGURE 12:**
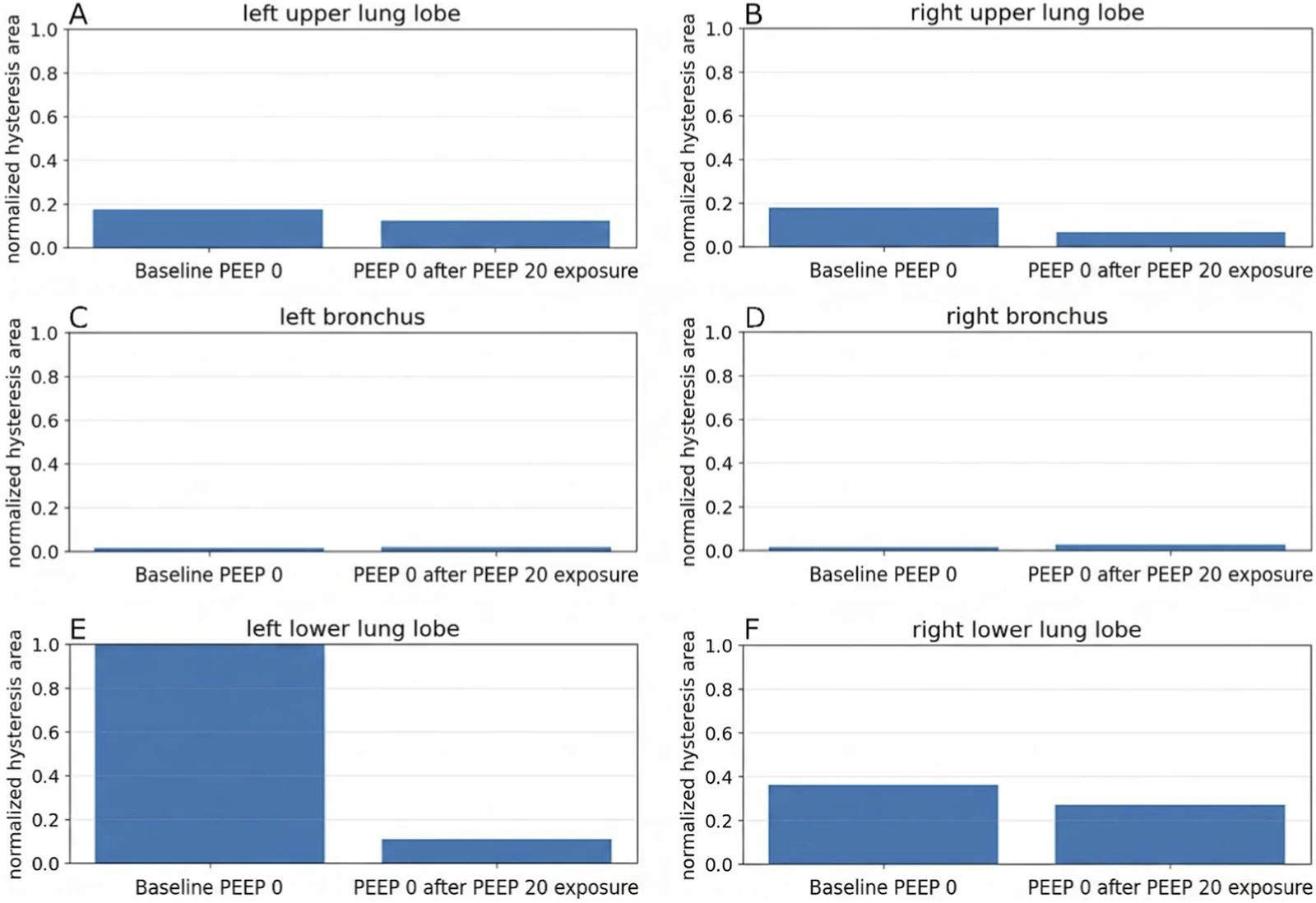
Comparison of normalized hysteresis areas at PEEP 0 before and after ventilation at PEEP 20. Each panel contrasts the normalized hysteresis area measured at baseline PEEP 0 (Baseline PEEP 0) with the value reassessed at PEEP 0 after the ventilation sequence up to PEEP 20 (PEEP 0 after PEEP 20 exposure). **(A)** Left upper lung lobe. **(B)** Right upper lung lobe. **(C)** Left bronchus. **(D)** Right bronchus. **(E)** Left lower lung lobe. **(F)** Right lower lung lobe. Hysteresis values remain low and mostly unchanged after ventilation in the bronchi **(C,D)** and upper lobes **(A,B)**, whereas the left lower lung lobe **(E)** shows a reduction in hysteresis after ventilation at PEEP 20, with the right lower lung lobe **(F)** showing a smaller decrease.

Peripheral lung regions demonstrated substantially reduced hysteresis after prior high PEEP ventilation. The largest effect was observed in the left lower lobe (Figure 13).

**FIGURE 13:**
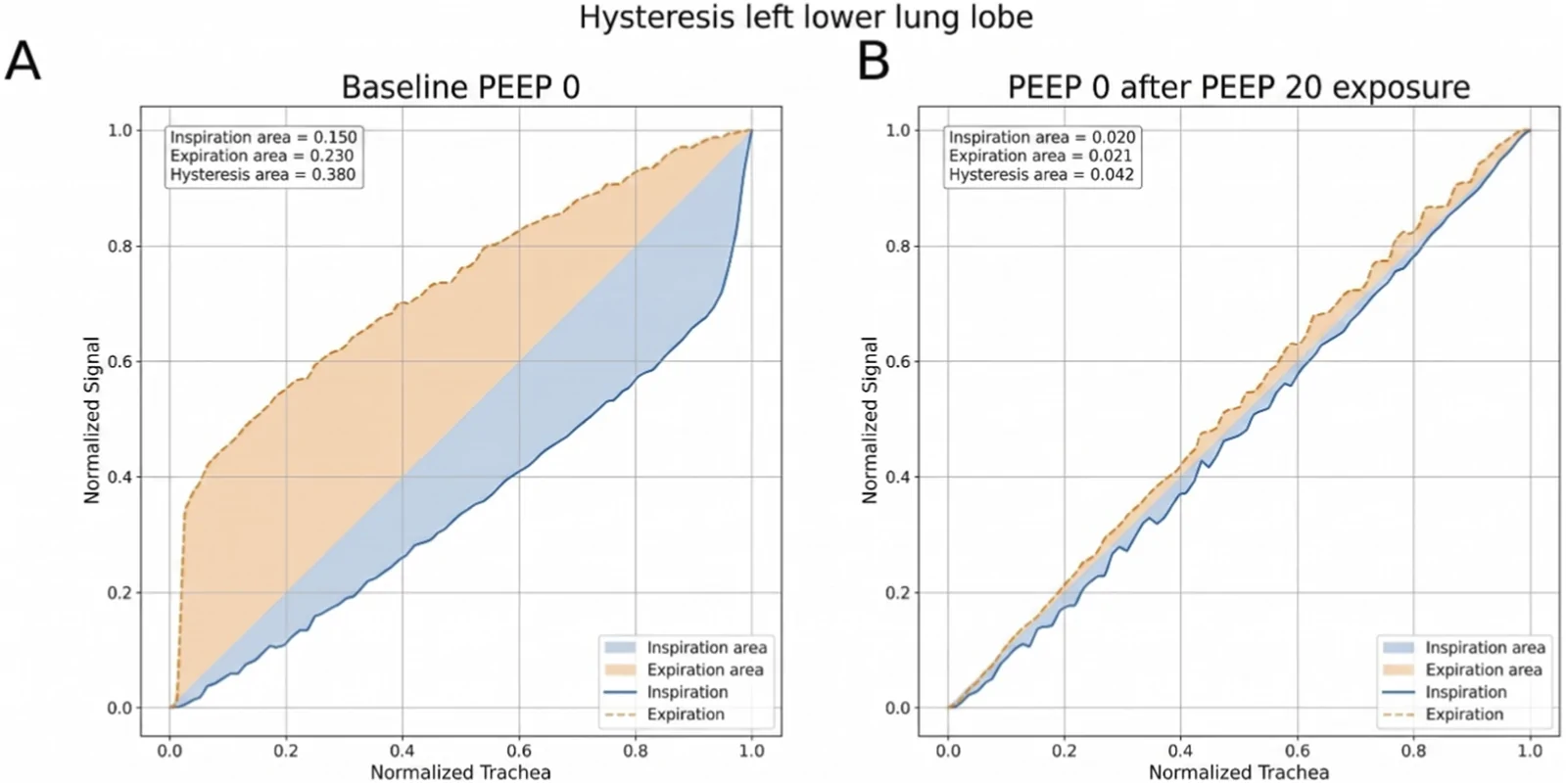
Hysteresis loops of the left lower lung lobe at PEEP 0 before and after ventilation at PEEP 20. Each panel shows the normalized regional pressure signal plotted against the normalized tracheal pressure during inspiration (blue, solid) and expiration (orange, dashed), with the inspiration area (blue) and expiration area (orange) shaded and the resulting hysteresis area indicated. **(A)** Baseline PEEP 0 before ventilation (Baseline PEEP 0; hysteresis area = 0.380). **(B)** PEEP 0 reassessed after the ventilation sequence up to PEEP 20 (PEEP 0 after PEEP 20 exposure; hysteresis area = 0.042).

Following prior exposure to PEEP 20 mbar, the inspiratory and expiratory pressure trajectories measured subsequently at PEEP 0 mbar were nearly superimposed, indicating reduced pressure-transmission hysteresis and more stable regional pressure behavior in the peripheral lung region (Figure 14). Importantly, both measurements compared in Figure 14 were obtained at PEEP 0 mbar. The first measurement was performed before exposure to elevated PEEP, whereas the subsequent PEEP 0 measurement was obtained after the lung had been ventilated at PEEP levels up to 20 mbar. The persistent change in regional pressure behavior after returning to PEEP 0 is consistent with a history-dependent mechanical effect and may reflect sustained opening or recruitment of previously unstable peripheral lung compartments.

**FIGURE 14:**
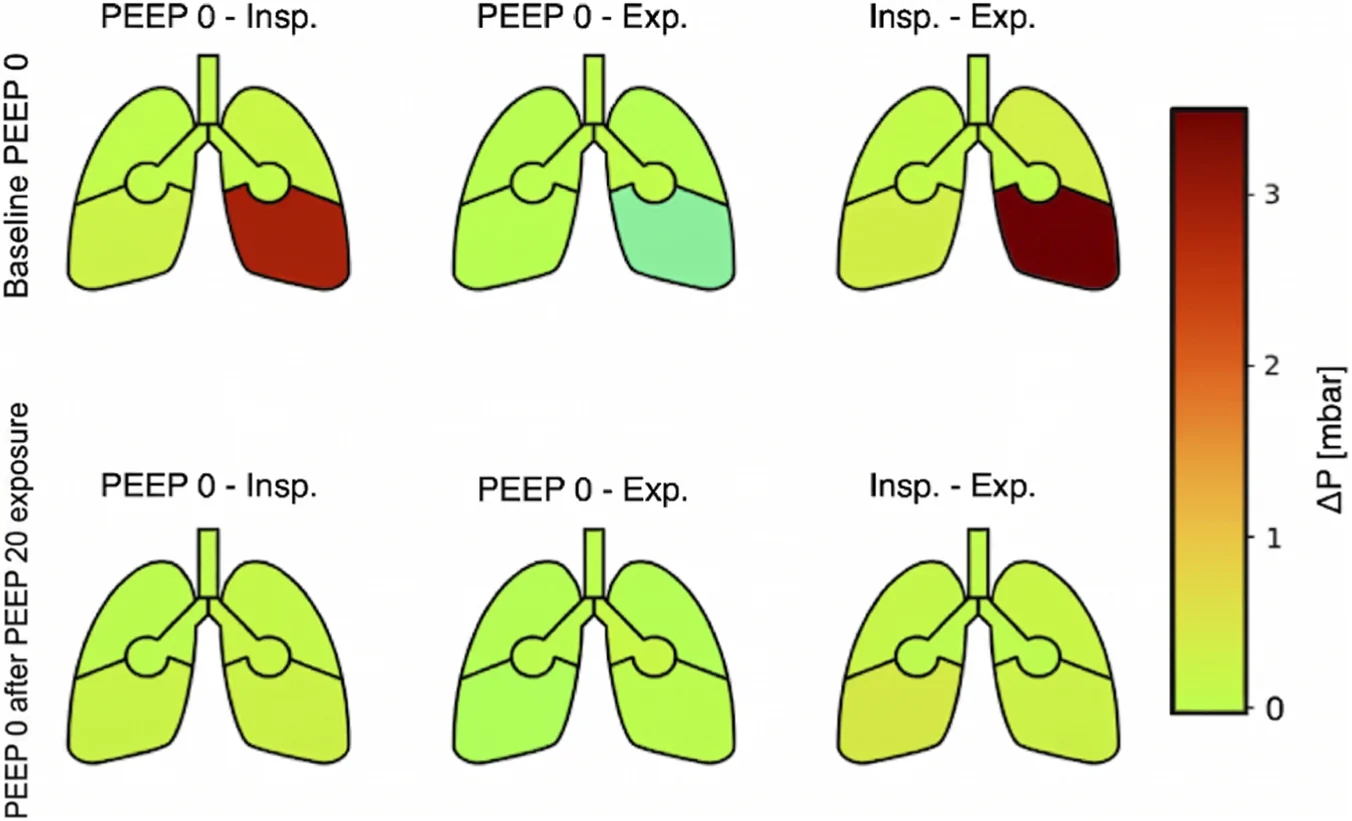
Regional trachea-to-region pressure differences at PEEP 0 before and after prior exposure to PEEP 20 mbar. The upper row shows the initial baseline measurement at PEEP 0, whereas the lower row shows the repeated PEEP 0 condition after completion of the ventilation sequence up to PEEP 20 mbar. Thus, both measurements were obtained at identical nominal PEEP while differing in the preceding mechanical history of the lung specimen.

The close alignment between the regional pressure data generated by the simulator and historical clinical observations validates the platform’s functional fidelity. The observed phenomenon of peripheral compartment instability at low PEEP, followed by stabilization at higher pressures, directly mirrors the macro-mechanical behaviors extensively documented in literature (Acute Respiratory Distress Syndrome Network et al., 2000; Gattinoni et al., 2016b). The fact that the simulator independently confirms these established concepts proves its technical accuracy and demonstrates that the physical boundary conditions within the thoracic chamber are correctly calibrated.

This agreement supports the technical plausibility of the measurement platform within the investigated specimen but does not constitute physiological validation of the simulator, proof of alveolar recruitment, or independent validation of the chamber boundary conditions. Confirmation against independent *ex vivo* or *in vivo* reference data remains necessary.

## 4 Discussion

The present study establishes a technical platform for synchronized measurement of regional intrapulmonary pressure during controlled mechanical ventilation. By combining biological airway anatomy with regional pressure sensing, synchronized airflow measurements, and a defined extrathoracic pressure environment, the system enables direct characterization of spatial pressure transmission within an *ex vivo* lung.

The study should be interpreted as a system-development and feasibility investigation rather than as physiological validation of a lung model. The platform enabled derivation of engineering metrics including regional pressure attenuation, pressure-flow indices, trachea-to-region regression parameters, pressure-trajectory hysteresis, Poincaré-derived dispersion, tidal volume, and dynamic system compliance.

Because all measurements were obtained from a single lung specimen and no independent *in vivo* reference standard was available, the results do not establish biological reproducibility or generalized physiological validity. Accordingly, the regional differences observed in this study are interpreted as specimen-specific technical observations.

Within the investigated specimen, static pressure transmission was relatively homogeneous, whereas dynamic pressure-trajectory behavior differed between central and peripheral regions. Increasing PEEP was associated with smaller regional pressure differences, lower pressure-trajectory hysteresis, and altered Poincaré-derived dispersion in several peripheral regions. These observations demonstrate that the platform can resolve changes in regional pressure behavior under different ventilator settings without implying a specific underlying alveolar mechanism.

This setting should be interpreted as a system-development and feasibility study rather than as a full physiological validation of a lung model. The results demonstrate that the platform can resolve regional pressure-transmission patterns and derive engineering metrics such as pressure attenuation, pressure-flow indices, trachea-to-region regression parameters, tidal volume, and dynamic system compliance.

Within the tested scope, the platform provides a controlled environment for technical investigations of ventilator-lung interaction. However, because the measurements were performed on a single specimen and without external *in vivo* benchmark data, the results do not establish biological reproducibility or general physiological validity.

Accordingly, all physiological observations presented in this study should be interpreted as specimen-specific findings obtained within a technical feasibility framework and should not be generalized to human lung physiology without further validation in multiple specimens and *in vivo* studies.

In the investigated specimen, peripheral lung regions, particularly the lower lobes, demonstrated increased mechanical energy dissipation and pressure variability under low PEEP conditions.

Within this experimental model, increasing PEEP was associated with more stable regional pressure behavior and reduced regional mechanical heterogeneity.

From a technical perspective, these findings indicate that global airway pressure measurements can be effectively complemented by local pressure assessments, and suggest that, in this specimen, recruitment state contributed to the observed heterogeneity in pressure distribution.

### 4.1 Regional pressure transmission

The thoracic chamber provided a defined engineering boundary condition with a nominal subatmospheric baseline of approximately −7 mbar. The recorded chamber-pressure waveform exhibited respiratory-cycle-dependent excursions and was not intended to reproduce the magnitude or temporal profile of physiological pleural pressure.

Regional inspiratory and expiratory pressures increased with increasing ventilator pressure across all measured lung regions. At most measurement sites, differences relative to tracheal pressure remained small, indicating close pressure coupling between the trachea and regional measurement locations within the investigated specimen.

The largest regional deviations occurred under low-PEEP conditions, particularly in peripheral regions. These observations demonstrate that the measurement platform is sensitive to regional differences in pressure transmission. Because the experiment was performed in a single *ex vivo* specimen and regional lung volume or alveolar opening was not measured directly, the observed differences cannot be attributed definitively to specific physiological mechanisms. However, their reduction with increasing PEEP and the persistent change observed after prior high-PEEP exposure are consistent with a history-dependent change in peripheral lung mechanics, potentially including opening or recruitment of previously unstable compartments.

### 4.2 Regional pressure-trajectory hysteresis and variability

Although absolute pressure transmission was relatively uniform within the investigated specimen, inspiratory and expiratory pressure trajectories differed between anatomical regions. Bronchial measurement sites showed only small separation between inspiratory and expiratory branches, whereas greater pressure-trajectory hysteresis was observed in peripheral regions, particularly at low PEEP.

The hysteresis metric used in the present study is a dimensionless index derived from normalized tracheal-to-regional pressure trajectories. It should therefore not be interpreted as mechanical work or energy dissipation and cannot be directly compared quantitatively with conventional pressure-volume hysteresis areas. Nevertheless, the direction of the observed PEEP-dependent changes can be compared qualitatively with previous experimental work.

In the left lower peripheral region, the normalized pressure-trajectory hysteresis area decreased from 0.380 at PEEP 0 mbar to 0.051 at PEEP 5 mbar and remained comparatively low at higher PEEP levels. Cheng et al., 1995 demonstrated that recruitment and derecruitment of lung units contribute substantially to pressure-volume hysteresis in excised lungs. Thus, although the absolute hysteresis areas cannot be compared because fundamentally different mechanical quantities were assessed, the marked reduction in pressure-trajectory hysteresis observed with increasing PEEP in the present experiment is qualitatively consistent with pressure-dependent changes in lung hysteresis described in previous experimental work. This comparison should be interpreted as agreement in the direction of the mechanical response rather than equivalence of the underlying mechanical parameter.

The regional trends are also qualitatively consistent with previous observations of pressure-volume hysteresis and mechanically heterogeneous behavior in *ex vivo* porcine lungs under different inflation conditions (Sattari et al., 2023) and with EIT-based observations of regional differences in opening and closing pressures and hysteresis in ARDS (Scaramuzzo et al., 2020). These studies, however, assessed pressure-volume behavior, tissue deformation, or impedance-derived parameters rather than direct regional intrapulmonary pressure trajectories. Accordingly, the present measurements provide a complementary engineering perspective on regional pressure transmission rather than a directly equivalent physiological metric.

Poincaré analysis provided a complementary description of regional pressure-state dispersion. Direct quantitative comparison with published values remains limited because comparable datasets of simultaneously acquired regional intrapulmonary pressures are scarce. (White et al., 2022), for example, applied Poincaré analysis to successive peak inspiratory pressure and PEEP values generated by mechanical ventilators and used SD1 and SD2 to characterize short- and long-term pressure variation. In contrast, the present analysis was based on regional intrapulmonary pressure measurements rather than global ventilator pressure signals. The pronounced Poincaré pattern observed in the left lower lung lobe at PEEP 0, characterized by a high SD1/SD2 ratio, indicates substantially greater relative pressure-state dispersion under the lowest PEEP condition. From a mechanical perspective, this finding is compatible with less stable and more heterogeneous regional pressure transmission in this peripheral compartment. However, because the Poincaré sequence was not analyzed as an uninterrupted temporal series and regional lung volume or alveolar recruitment was not measured directly, ΔSD should not be interpreted as a conventional temporal variability metric or as direct evidence of recruitment or derecruitment.

Taken together, the greater peripheral pressure-trajectory hysteresis and pressure-state dispersion observed at low PEEP, and their marked reduction at PEEP ≥ 5 mbar, are broadly compatible with previously described spatially heterogeneous and pressure-dependent lung mechanics. In the left lower lung lobe, the reduction in the Poincaré pattern at higher PEEP occurred in parallel with the decrease in pressure-trajectory hysteresis, suggesting pressure-dependent stabilization of regional mechanical behavior. Potential mechanisms may include changes in regional compliance, airway or compartment opening and closing, or altered mechanical coupling within the peripheral lung. This qualitative agreement supports the mechanical plausibility of the observed regional pressure behavior, but it does not demonstrate alveolar recruitment, sustained alveolar opening, or a general physiological mechanism. The findings should therefore be interpreted as specimen-specific observations obtained within a technical feasibility framework.

### 4.3 Recruitment effects after high PEEP ventilation

In this study, an additional experiment evaluated whether prior exposure to high PEEP produced a persistent change in regional mechanical behavior within the lung model. After ventilation at PEEP 20 mbar, PEEP was returned to 0 mbar, and regional pressure behavior was compared with that observed during the initial PEEP 0 condition.

The analysis revealed a marked reduction in pressure-trajectory hysteresis after prior high-PEEP ventilation, particularly in the left lower peripheral region, where the normalized hysteresis area decreased from 0.380 to 0.042 at nominally identical PEEP 0 conditions (Figures 12,13). Inspiratory and expiratory pressure trajectories became substantially more aligned, indicating reduced phase-dependent pressure-transmission hysteresis. This dependence on prior high-PEEP exposure is consistent with observations in excised lungs showing that previous inflation and end-expiratory pressure can influence subsequent pressure-volume behavior (Cheng et al., 1995; Horie and Hildebrandt, 1973). The persistence of this altered pressure behavior after PEEP was returned to 0 mbar is consistent with a history-dependent mechanical effect and may reflect persistent opening or recruitment of previously unstable peripheral lung compartments.

### 4.4 Integrated interpretation

Taken The combined analysis of regional absolute pressure, trachea-to-region pressure differences, pressure-trajectory hysteresis, and Poincaré-derived dispersion demonstrates that the platform can distinguish static from dynamic aspects of regional pressure transmission.

Within the investigated specimen, absolute pressure transmission remained comparatively homogeneous, whereas dynamic pressure behavior varied more clearly between central and peripheral regions and changed with PEEP. The return to PEEP 0 after prior high-PEEP exposure further demonstrated that regional pressure trajectories may depend on the preceding mechanical state of the specimen.

These observations are qualitatively consistent with established concepts of history-dependent lung mechanics and with experimental studies showing PEEP-dependent changes in lung hysteresis (Cheng et al., 1995; Zheng, 2023). However, the present study measures regional intrapulmonary pressure rather than alveolar opening, regional lung volume, or tissue strain. The findings therefore support the technical sensitivity and physiological plausibility of the measurement approach but do not establish a general mechanism of recruitment or derecruitment.

These observations suggest that, in this specimen, regional pressure behavior depended not only on instantaneous ventilator settings but also on volume and recruitment history. The influence of recruitment history observed in the present study is also in agreement with current concepts of respiratory mechanics. Recent reviews describe lung recruitment as a pressure- and history-dependent process in which previous inflation and end-expiratory pressure substantially influence subsequent pressure–volume behaviour and regional mechanical heterogeneity. Consequently, the present observations are consistent with established physiological concepts, although confirmation in additional specimens will be required (Zheng, 2023).

### 4.5 Limitations

Several limitations should be considered when interpreting these findings.

First, the experimental setup represents an *ex vivo* lung model and does not reproduce the full physiological environment of the intact thorax. The model lacks pulmonary perfusion, active cellular and metabolic processes, respiratory-muscle activity, and physiological chest-wall mechanics. In addition, a preserved commercially available porcine lung was used. Although preservation maintains gross anatomical structure and elastic properties, preservation-related alterations in tissue compliance, viscoelasticity, and regional mechanical behavior cannot be excluded.

Second, the lung was positioned within an air-filled chamber and supported by a solid surface. In contrast to the native thorax, the model lacks a fluid-coupled pleural interface and continuous pleural contact. The absence of pleural fluid and physiological pleural coupling may increase gravity-dependent deformation, regional compression, or partial collapse and may therefore influence regional pressure transmission and mechanical behavior. Furthermore, the chamber provided a defined engineering boundary condition rather than reproducing the magnitude, spatial gradients, or temporal profile of physiological pleural pressure.

Third, regional pressure measurements were obtained at discrete anatomical sites and therefore cannot capture the continuous spatial distribution of pressure throughout the lung. Cannula placement was adapted to local airway anatomy and did not use a uniform insertion depth. Although channel patency and signal consistency were verified, small differences in local cannula position may have contributed to regional measurement variability.

Fourth, regional lung volume, alveolar opening, and tissue deformation were not measured directly. Consequently, PEEP-dependent changes in regional pressure behavior and the persistent changes observed after prior high-PEEP exposure cannot be interpreted as direct evidence of alveolar recruitment or sustained alveolar opening. Such mechanisms remain plausible interpretations that require confirmation using complementary imaging or regional volume measurements.

Fifth, the pressure-trajectory hysteresis metric is based on normalized tracheal-to-regional pressure relationships and cannot be interpreted as mechanical work or energy dissipation or directly compared quantitatively with conventional pressure-volume hysteresis. Similarly, the Poincaré-derived metrics were used as exploratory descriptors of pressure-state dispersion rather than as validated physiological biomarkers.

Finally, all experiments were performed using a single lung specimen. Repeated measurements therefore quantify technical within-specimen repeatability but not biological reproducibility. The observed regional pressure patterns and PEEP-dependent changes should consequently be regarded as specimen-specific technical findings. Confirmation in multiple specimens and validation against independent *ex vivo* or *in vivo* reference measurements will be required before broader physiological conclusions can be drawn.

## 5 Conclusion

This study demonstrates the technical feasibility of a biomimetic *ex vivo* thorax–lung simulator for synchronized regional intrapulmonary pressure measurements during controlled mechanical ventilation.

The measurement system enabled stable multichannel pressure acquisition, synchronization of independently recorded pressure and airflow signals, reconstruction of tidal volume and dynamic system compliance, and reproducible within-specimen measurements across different PEEP conditions. Repeated measurements showed high short-term technical repeatability, with repeated median regional pressure values differing by approximately 0.02 mbar. Chamber-pressure monitoring demonstrated a reproducible subatmospheric baseline of approximately −7 mbar with superimposed respiratory-cycle-dependent pressure excursions.

Within the investigated specimen, absolute pressure transmission was comparatively homogeneous, whereas dynamic pressure behavior differed between central and peripheral measurement regions. Peripheral regions showed greater pressure-trajectory hysteresis and Poincaré-derived pressure-state dispersion at low PEEP, both of which decreased under higher PEEP conditions. Following prior exposure to PEEP 20 mbar, altered regional pressure behavior persisted after PEEP was returned to 0 mbar, indicating a history-dependent change in peripheral lung mechanics. This finding may be consistent with persistent opening or recruitment of previously unstable peripheral compartments, although alveolar recruitment was not measured directly.

The observed pressure-dependent and history-dependent changes are qualitatively consistent with previous experimental observations of lung hysteresis and recruitment-related mechanical behavior (Cheng et al., 1995; Zheng, 2023). However, because the present study was performed using a single preserved *ex vivo* lung specimen and without an independent physiological reference standard, these observations should be interpreted as specimen-specific technical findings rather than evidence of a general physiological mechanism.

The platform therefore provides a technical and methodological foundation for future studies involving multiple biological specimens, complementary regional imaging or volume measurements, disease models, and validation against independent *ex vivo* or *in vivo* reference data.

## Data Availability

The original contributions presented in the study are included in the article/supplementary material, further inquiries can be directed to the corresponding author.
